# A rare codon-based translational program of cell proliferation

**DOI:** 10.1186/s13059-020-1943-5

**Published:** 2020-02-27

**Authors:** Joao C. Guimaraes, Nitish Mittal, Alexandra Gnann, Dominik Jedlinski, Andrea Riba, Katarzyna Buczak, Alexander Schmidt, Mihaela Zavolan

**Affiliations:** 1 Computational and Systems Biology, Biozentrum University of Basel, Klingelbergstrasse 50-70, 4056 Basel, Switzerland; 2 Department of Biomedicine, University of Basel/University Hospital Basel, Hebelstrasse 20, 4031 Basel, Switzerland; 3 Institut de Génétique et de Biologie Moléculaire et Cellulaire, Université de Strasbourg, 1 rue Laurent Fries, 67404 Illkirch CEDEX, France; 4 Proteomics Core Facility, Biozentrum University of Basel, Klingelbergstrasse 50-70, 4056 Basel, Switzerland

**Keywords:** Cell proliferation, Translation control, Codon usage, Rare codons, Translation elongation, tRNA pools

## Abstract

**Background:**

The speed of translation elongation is primarily determined by the abundance of tRNAs. Thus, the codon usage influences the rate with which individual mRNAs are translated. As the nature of tRNA pools and modifications can vary across biological conditions, codon elongation rates may also vary, leading to fluctuations in the protein production from individual mRNAs. Although it has been observed that functionally related mRNAs exhibit similar codon usage, presumably to provide an effective way to coordinate expression of multiple proteins, experimental evidence for codon-mediated translation efficiency modulation of functionally related mRNAs in specific conditions is scarce and the associated mechanisms are still debated.

**Results:**

Here, we reveal that mRNAs whose expression increases during cell proliferation are enriched in rare codons, poorly adapted to tRNA pools. Ribosome occupancy profiling and proteomics measurements show that upon increased cell proliferation, transcripts enriched in rare codons undergo a higher translation boost than transcripts with common codons. Re-coding of a fluorescent reporter with rare codons increased protein output by ~ 30% relative to a reporter re-coded with common codons. Although the translation capacity of proliferating cells was higher compared to resting cells, we did not find evidence for the regulation of individual tRNAs. Among the models that were proposed so far to account for codon-mediated translational regulation upon changing conditions, the one that seems most consistent with our data involves a global upregulation of ready-to-translate tRNAs, which we show can lead to a higher increase in the elongation velocity at rare codons compared to common codons.

**Conclusions:**

We propose that the alleviation of translation bottlenecks in rapidly dividing cells enables preferential upregulation of pro-proliferation proteins, encoded by mRNAs that are enriched in rare codons.

## Introduction

Due to the redundancy of the genetic code, the same protein can be encoded in many distinct mRNA sequences. Synonymous codons are not uniformly represented in the transcriptome; the observed codon bias having coevolved with transfer RNA (tRNA) abundances under selection for translation accuracy and efficiency, among others [1–6]. Although initiation is the primary rate-limiting step of translation [3, 7], the rate at which the ribosome elongates the polypeptide chain also modulates the protein output, as elongation-induced “traffic jams” may affect the initiation rate or lead to abortion of translation [8]. The decoding rate of synonymous codons varies widely, depending primarily on the abundance of cognate tRNAs [9–12]. Common codons are recognized by abundant tRNAs and are more efficiently translated than rare codons, whose frequency of occurrence in the transcriptome is low. In addition to modulating protein output, the choice among synonymous codons has been shown to influence transcript stability [13–15], protein folding [11, 16–19], and function [20, 21].

Recent studies have highlighted examples where the abundance and modifications of individual tRNAs are adjusted to match the codon usage of functionally related genes that are expressed specifically in individual human tissues [22, 23], during cell differentiation [24–26] and cellular stress [27], or in tumors [28]. In particular, tRNAs that were found induced in proliferative cells generally decode codons that are over-represented in pro-proliferative mRNAs [24]. This suggested that tRNA pools fluctuate between cellular states such as proliferation and differentiation to match the “codon demand” of the corresponding transcriptomes and presumably enhance translation efficiency. However, subsequent studies argued that the codon bias of specific classes of mRNAs reflects primarily local sequence evolution due to meiotic expression and recombination, and not selection for translation [29, 30]. Furthermore, perturbations of the protein synthesis apparatus, whether due to altered ribosome composition [31, 32] or ribosome levels [33, 34], altered interactions of translation initiation factors [35–37], or post-translational modifications in ribosomal proteins [38], can specifically impact the translation of subsets of mRNAs. The observation that cell cycle-controlled genes are enriched in non-optimal codons, presumably supporting their coordinated oscillatory expression, was especially intriguing [39]. However, whether and how codon signatures of individual mRNAs lead to differences in their translation efficiency in resting compared to proliferating cells is still unclear.

By measuring translation efficiencies transcriptome-wide in cells that proliferate at different rates, we here show that pro-proliferative mRNAs, which are enriched in rare codons, undergo a stronger translation boost in rapidly dividing cells than mRNAs encoded with common codons. Reporter constructs re-coded with either rare or common codons exhibit the same behavior. We do not find differences in the expression of individual tRNAs between resting and proliferating cells. Of the models proposed to relate translational changes to variation in tRNA pools, the one that remains consistent with our data is that the elongation velocity at rare codons improves more than that of common codons if global ready-to-translate tRNA pools increase during cell proliferation. Taken together, our data suggest that changes in a general resource, in this case, translation elongation capacity, can concertedly modulate a restricted, functionally related set of molecular targets to control specific phenotypes.

## Results

### Proliferation-induced mRNAs are enriched in rare codons

**Fig. 1:**
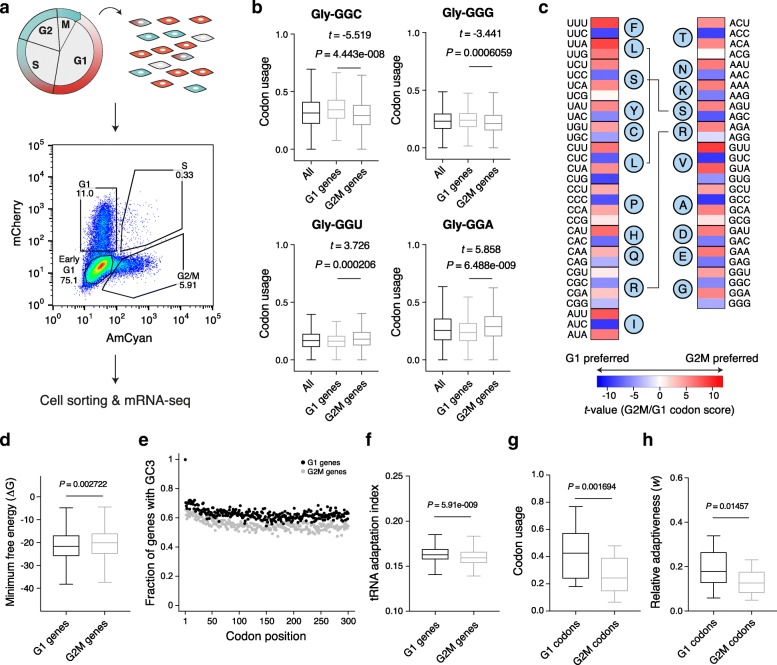
mRNAs required for cell proliferation are enriched in rare codons and are poorly adapted to tRNA pools. **a** NIH-3T3 cells with a stably integrated FUCCI system were sorted according to the cell cycle phase, and the corresponding transcriptomes were profiled by mRNA sequencing. The scatter plot shows the expression of AmCyan and mCherry in individual cells. The percentage of cells in each cell cycle phase is depicted. **b** Boxplots showing relative usage of synonymous glycine-encoding codons across all genes, or in genes expressed preferentially in either G1 or G2/M phases. Shown are the *t* and *P* values determined by the unpaired, two-tailed, Welch *t* test comparing the codon usage of G1 genes to that of G2/M genes. **c** Heatmap showing the *t* value (G2M/G1 codon score) for all codons. Codons that are preferentially used in G2/M genes have positive codon scores, whereas codons enriched in G1 genes have negative codon scores. The amino acid encoded by each codon is shown with a single-letter code: F, phenylalanine; L, leucine; S, serine; Y, tyrosine; C, cysteine; P, proline; H, histidine; Q, glutamine; R, arginine; I, isoleucine; T, threonine; N, asparagine; K, lysine; V, valine; A, alanine; D, aspartate; E, glutamate; G, glycine. **d** Boxplots showing the distribution of the minimum free energy (MFE) for RNA folding of the translation initiation region ([− 40, + 40] nucleotides with respect to the start codon) of G1- and G2/M-enriched mRNAs. Shown is the *P* value determined by the non-parametric Mann-Whitney *U* test. **e** Scatter plot depicting the fraction of G1- and G2/M-enriched mRNAs with G/C at the third nucleotide position in the first 300 codons. **f** Boxplots showing the distribution of tRNA adaptation index for G1- and G2/M-enriched mRNAs. Shown is the *P* value determined by the non-parametric Mann-Whitney *U* test. **g**, **h** Boxplots showing the distribution of the transcriptome-wide usage (weighted by transcript expression (**g)** or relative adaptiveness to tRNA pools (**h**) of the codons that are preferentially used (|*t* value|> 3) in genes associated with a specific cell cycle phase (G1, *n* = 22 codons, G2/M, *n* = 29 codons). Shown are the *P* values determined by the non-parametric Mann-Whitney *U* test. All boxes extend from the 25th to 75th percentiles (inter-quartile range (IQR)), horizontal lines represent the median, and whiskers indicate the lowest and highest datum within 1.5*IQR from the lower and upper quartiles, respectively

To determine the codon usage in different proliferative states, we tracked the cell cycle progression of mouse embryonic fibroblast NIH-3T3 cells with the FUCCI system of genetically encoded fluorescent probes [40]. By sorting cells in the G1 and G2/M cell cycle phases and profiling their transcriptomes (Fig. 1a), we found that mRNAs encoding proteins that are involved in cell adhesion and specification were preferentially expressed in G1 cells, whereas mRNAs encoding proteins responsible for DNA replication and cell division were upregulated in G2/M cells (Additional file 1: Figure S1A, B). Although the codon usage of the entire transcriptome remained similar between the two cell cycle phases, the mRNAs whose expression was higher in the G2/M relative to G1 phase had a markedly distinct codon usage (Fig. 1b, Additional file 1: Figure S1C and Additional file 2: Table S1), consistent with previous reports [24, 39]. We used the *t* test to quantify these differences; for any codon, a positive or negative *t* value (G2M/G1 codon score, Fig. 1b, c and Additional file 3: Table S2) reflects its preferential use in mRNAs with higher expression in the G2/M or G1 phase, respectively. mRNAs enriched in the G2/M phase exhibited a strong preference for codons whose third nucleotide was an adenine or uridine (A/U), whereas G1-enriched mRNAs used codons ending in guanine or cytosine (G/C) (Fig. 1c). The use of A/U-rich codons at the 5′ end of coding regions has been associated with a reduced propensity to form RNA secondary structures, which hinder translation initiation [41–46]. Although the translation initiation region of G2/M mRNAs indeed had significantly higher predicted free energy of folding than the corresponding region of G1 mRNAs (thus “weaker” RNA structure, Fig. 1d), A/U-rich codons were preferentially used throughout the coding sequence of G2/M mRNAs, suggesting that the impact of these codons goes beyond translation initiation (Fig. 1e). The genes induced in the G2/M phase are significantly less adapted to the tRNA pools computationally inferred from gene copy numbers (Fig. 1f); individual codons that are over-represented in these genes (*t* value > 3) are less frequently used in the transcriptome (thus “rare codons,” Fig. 1g) and are decoded by less abundant tRNAs (Fig. 1h).

### Codon usage is correlated with tissue proliferative capacity

To further validate the association between codon choice and the proliferation state of cells, we compared the codon usage of genes with higher than average expression in each of 15 different mouse tissues with the codon usage of G1- and G2/M-enriched genes (Additional file 1: Figure S2A). Remarkably, genes that are preferentially expressed in tissues with high proliferative potential (such as testis and thymus) shared the codon usage of genes enriched in the G2/M phase, whereas genes with specific expression in tissues with low proliferative potential (such as lung or cortex) shared the codon bias of G1-enriched genes. The overlap of tissue-specific genes with those specifically expressed in different cell cycle phases was very small (Additional file 1: Figure S2B, C) demonstrating that the choice between rare and common codons is not restricted to genes whose expression changes between different phases of the cell cycle, but extends to genes that are specifically expressed in individual tissues.

### Translation boost of mRNAs enriched in rare codons during proliferation

**Fig. 2:**
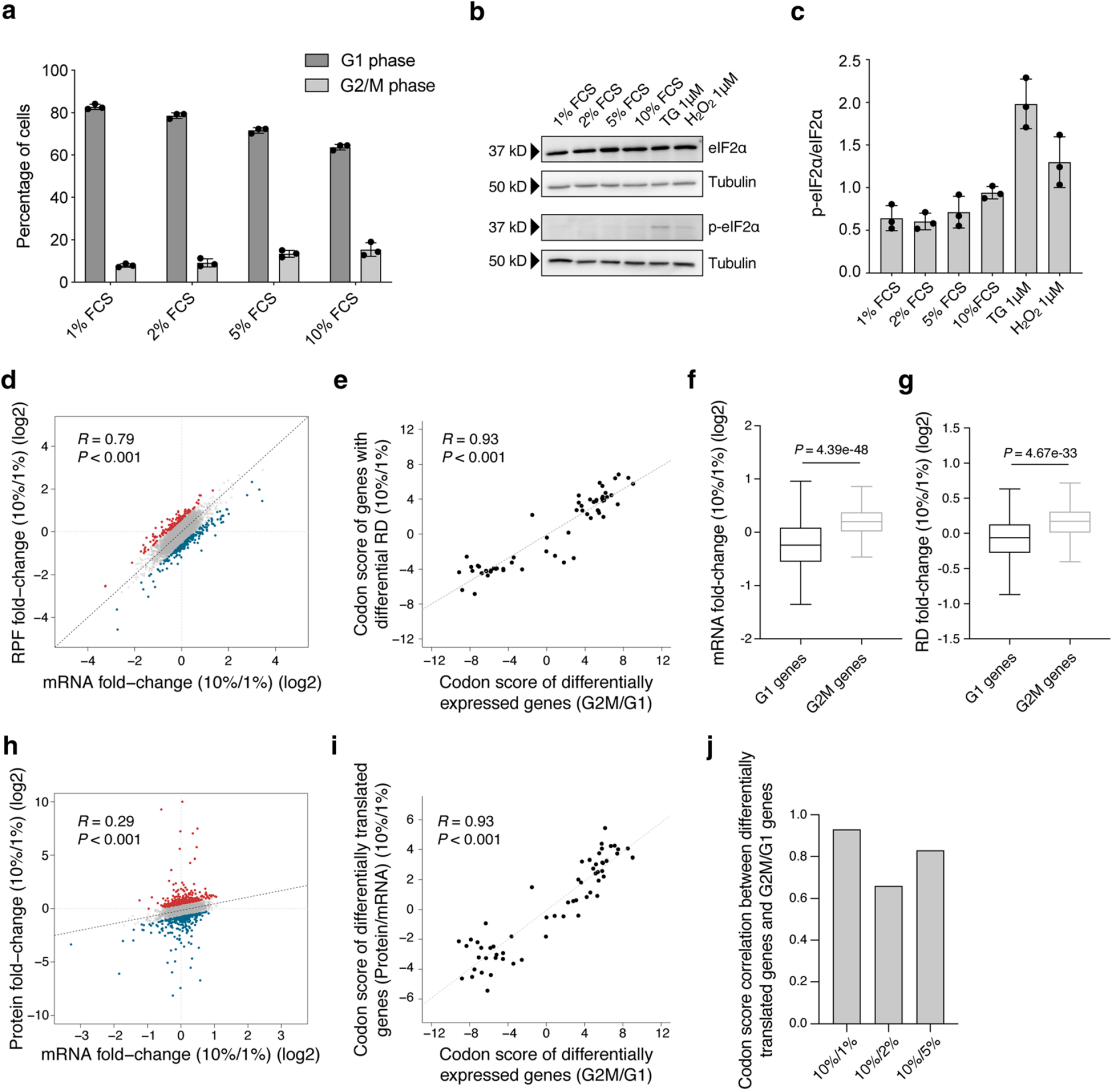
Distinct codon usages are associated with transcriptome-wide translation efficiencies in different proliferative states. **a** Mean (± s.e.m.) percentage of wild-type NIH-3T3 cells in the G1 and G2/M phases of the cell cycle, as determined by Hoechst staining for DNA content, for cells grown in either 1%, 2%, 5%, or 10% FCS (*n* = 3). **b** Representative western blot depicting the levels of phosphorylated eIF2α (p-eIF2α), total eIF2α, and the loading control tubulin, for cells growing in either 1%, 2%, 5%, or 10% FCS, as well as in two control conditions triggering stress responses (thapsigargin (TG) and hydrogen peroxide (H_2_O_2_)). **c** Mean (± s.e.m.) level of p-eIF2α normalized by total eIF2α estimated from western blots, for cells growing in either 1%, 2%, 5%, or 10% FCS, as well as in two control conditions triggering stress responses (thapsigargin (TG) and hydrogen peroxide (H_2_O_2_)) (*n* = 3). **d** Scatter plot of the log2 fold changes in mRNA and ribosome protected fragments (RPF) in cells grown in 10% relative to 1% FCS. Shown are the mean values computed for each transcript from mRNA (*n* = 4) and RPF (*n* = 3) replicates. Transcripts upregulated and downregulated at ribosome density level (RD, defined as RPF_fold change_/mRNA_fold change_), i.e., changed more than 50% in either direction, are shown in red and blue, respectively. Shown are the Pearson correlation coefficient and respective *P* value. The dashed line indicates equal change in mRNA and RPF levels. **e** Scatter plot of per codon scores among genes that are differentially expressed between G2/M and G1 cell cycle phases and genes with differential ribosome density (RD, defined as RPF_fold change_/mRNA_fold change_) when cells are grown in 10% relative to 1% FCS. Shown are the Pearson correlation coefficient and respective *P* value. The dashed line indicates the linear regression between the two estimates. Boxplots showing the distribution of log2 fold changes in mRNA (**f**) and RD (**g**) between cells grown in 10% relative to 1% FCS, for genes with preferential expression in the G1 and G2/M cell cycle phases. Shown are the *P* values determined by the non-parametric Mann-Whitney *U* test. Boxes extend from the 25th to 75th percentiles (inter-quartile range (IQR)), horizontal lines represent the median, and whiskers indicate the lowest and highest datum within 1.5*IQR from the lower and upper quartiles, respectively. **h** Scatter plot of the log2 fold changes in mRNA and protein levels in cells grown in 10% relative to 1% FCS. Shown are the mean values computed for each transcript/protein from mRNA-seq (*n* = 3) and proteomics (*n* = 3) data. Transcripts upregulated and downregulated at translational level (defined as the residuals of the linear regression between Protein_fold change_ and mRNA_fold change_), i.e., changed more than 50% in either direction, are shown in red and blue, respectively. Shown are also the Pearson correlation coefficient and respective *P* value. The dashed line indicates the linear regression between the two measurements. **i** Scatter plot of per codon scores among genes that are differentially expressed between G2/M and G1 cell cycle phases and differential translated genes (see above) when cells are grown in 10% relative to 1% FCS. Shown are also the Pearson correlation coefficient and respective *P* value. The dashed line indicates the linear regression between the two estimates. **j** Correlation between codon scores of genes that are differentially expressed between G2/M and G1 cell cycle phases and differential translated genes (see above) estimated from cells growing in 10% relative to either 1%, 2%, or 5% FCS

As rare codons have been reported to reduce the rate of translation elongation [11], we sought to determine the impact of codon usage on protein synthesis rates in cells growing at different proliferation rates. We induced NIH-3T3 cells to proliferate at different rates by culturing them in either 1%, 2%, 5%, or 10% fetal calf serum (FCS) (Fig. 2a and Additional file 1: Figure S3A). By monitoring the phosphorylation status of eIF2α (p-eIF2α) [47], we confirmed that growing cells with lower serum concentrations did not trigger a stress response (Kruskal-Wallis test *P* = 0.1027, Fig. 2b, c). We further established that cell viability was not compromised under these conditions (Additional file 1: Figure S3B, C). Thus, to study the effect of cell proliferation on mRNA-specific translation regulation, we analyzed cells growing in either 1% or 10% FCS. We first profiled the ribosome occupancy and relative abundance of all expressed mRNAs (Additional file 1: Figure S4 and Additional file 4: Table S3) and identified a few hundred transcripts whose ribosome density (i.e., ribosome occupancy fold change normalized by mRNA fold change) changed more than 50% in either direction between the two conditions (Fig. 2d, red and blue dots). The codon scores of mRNAs exhibiting differential ribosome density (RD) under distinct proliferative conditions were strikingly similar to that observed when comparing G1- and G2/M-induced mRNAs (Fig. 2e, Pearson correlation coefficient *R* = 0.93, *P* < 0.001). Attesting to the significance of this correlation, the codon scores obtained by comparing two random subsets of genes showed much more modest correlations with the G2M/G1 codon scores (Additional file 1: Figure S5A). Finally, this association was also evident when considering those genes whose RD is changed but not the mRNA level (Additional file 1: Figure S5B, C).

Consistent with a higher proportion of dividing cells in 10% FCS, we observed increased expression of mRNAs previously found enriched in the G2/M phase using the FUCCI system (Fig. 2f). Remarkably though, not only was the level of these mRNAs higher, but also their ribosome density compared to that of G1-enriched mRNAs (Fig. 2g). We then pursued the quantification of protein levels genome-wide to validate if mRNAs more densely occupied by ribosomes also exhibited elevated protein synthesis rates (Additional file 5: Table S4). We identified a few hundred transcripts whose translation efficiency (i.e., protein fold change normalized by mRNA fold change) changed more than 50% in either direction between cells cultured in 1% and 10% FCS (Fig. 2h, red and blue dots). We confirmed that mRNAs previously identified with differential RD also displayed a corresponding significant change in translation efficiency (Additional file 1: Figure S5D). In agreement with the ribosome density data, the codon scores of differentially translated mRNAs were strongly correlated to those of genes that were differentially expressed between G1 and G2/M phases (Fig. 2i, Pearson correlation coefficient *R* = 0.93, *P* < 0.001). Importantly, we obtained equally strong associations between codon scores when comparing cells growing in either 2% or 5% with cells growing in 10% (Fig. 2j and Additional file 1: Figure S5E, F), ruling out the possibility that our observations are exclusive to cells growing at very low serum concentrations. Altogether, these results indicate that mRNAs enriched in rare codons exhibit enhanced translation in rapidly proliferating cells relative to cells that proliferate slowly, compared to transcripts that do not share this codon bias.

### Rare codons confer a preferential boost in the protein output of fluorescent reporters

**Fig. 3:**
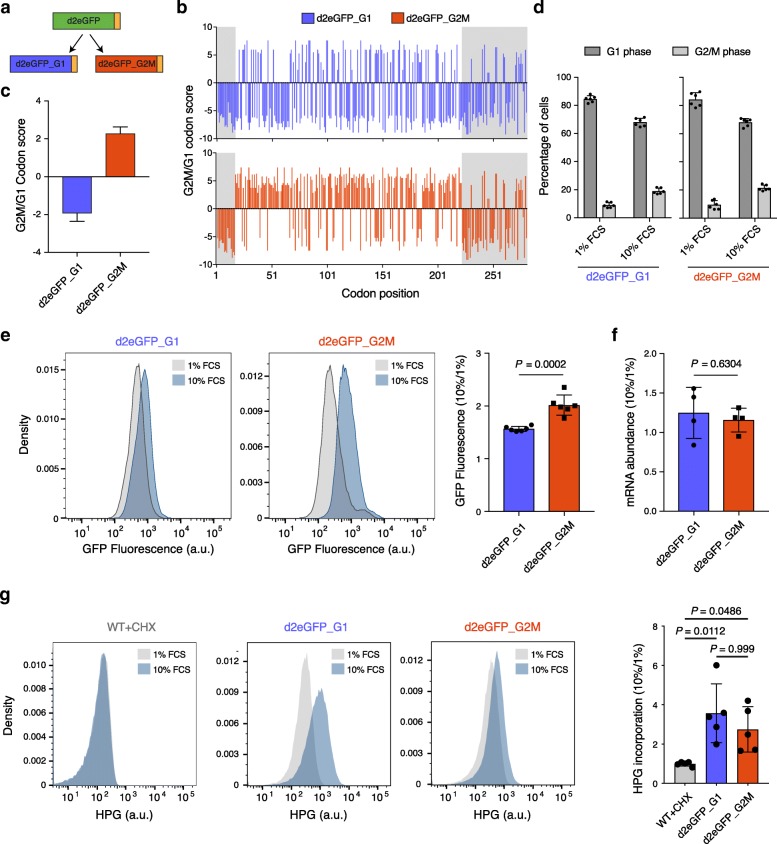
Rare codons confer increased translation efficiency in rapidly dividing cells. **a** Starting from the rapid turnover rate protein d2eGFP [48], we designed two fluorescent reporters, encoded with codons that are more frequently used in either G1- or G2/M-enriched mRNAs. Yellow box corresponds to the PEST sequence attached to the C-terminal region of the eGFP protein. **b** G2M/G1 codon score (Fig. 1c) for each codon of the d2eGFP_G1 (top) and d2eGFP_G2M (bottom) reporters. The first and last 17 codons (51 nucleotides) of eGFP, as well as the PEST sequence, were left unchanged (gray regions) to avoid effects on translation initiation and termination. **c** Mean (± s.e.m.) G2M/G1 codon score for the re-coded region in the 2 fluorescent reporters. **d** Mean (± s.e.m.) percentage of cells in the G1 and G2/M phases of the cell cycle, as determined by Hoechst staining for DNA content, for the d2eGFP_G1 and d2eGFP_G2M cell lines grown in either 1% or 10% FCS (*n* = 6). **e** Representative result of the GFP fluorescence intensity measurement by flow cytometry in the 2 cell lines grown in either 1% or 10% FCS (left). Mean (± s.e.m.) ratio of fluorescence intensity for cells grown in 10% relative to 1% FCS for the two reporters (*n* = 6) (right). Shown is the *P* value determined by the non-parametric Mann-Whitney *U* test. **f** Mean (± s.e.m.) ratio of transcript abundance, as measured by qRT-PCR, for cells grown in 10% relative to 1% FCS for the 2 reporter cell lines (*n* = 4) (right). Shown is the *P* value determined by the non-parametric Mann-Whitney *U* test. **g** Representative result of HPG incorporation measurement (a.u., arbitrary units) by flow cytometry in the 2 cell lines growing in either 1% or 10% FCS (left). Quantification of the mean (± s.e.m.) ratio of HPG incorporation in 10% compared to 1% FCS for the 2 reporter cell lines (*n* = 5) (right). For control, we also incubated wild-type NIH-3T3 cells with the translation inhibitor cycloheximide (CHX). Shown are the *P* values determined by Dunn’s multiple comparisons test post hoc and the non-parametric Kruskal-Wallis test (*P* = 0.0018)

To validate that the codon choice directly affects the translation efficiency, we re-coded a fluorescent protein with rapid turnover rate, d2eGFP [48], generating two reporters that conformed to the codon bias of genes that are preferentially expressed in the two different cell cycle phases (d2eGFP_G1 and d2eGFP_G2M, Fig. 3a–c). The 5′- and 3-terminal regions of these reporters were left unchanged, so as to minimally interfere with translation initiation and termination (Fig. 3b, gray regions). We then generated stable cell lines, each expressing one of the reporters, and cultured them in either 1% or 10% FCS to induce distinct proliferation rates (Fig. 3d and Additional file 1: Figure S6). Fluorescence intensity measurements based on flow cytometry revealed that the relative expression of the d2eGFP_G2M construct was significantly higher than that of the d2eGFP_G1 construct in cells grown in 10% compared to 1% FCS (Fig. 3e), while the relative mRNA levels of the two reporters remained similar (Fig. 3f). Importantly, the observed changes did not result from differences in total protein synthesis rates of the two cell lines (Fig. 3g), as these underwent a similar change when the cell lines were grown in 10% relative to 1% FCS, as shown by the incorporation of the methionine analog l-homopropargylglycine (HPG) in newly synthesized proteins with a fluorimetric assay. These data show that a reporter construct enriched in rare codons was able to produce ~ 30% more protein compared to a synonymous construct enriched in common codons, when cells were induced to proliferate rapidly. Moreover, the observed translational boost should not be due to a direct change in the initiation, because the translation initiation regions of the two constructs were identical, thus rather originating from different elongation rates of reporter mRNAs.

### Most current models of tRNA pool variations do not appear to underlie the observed translational changes

**Fig. 4:**
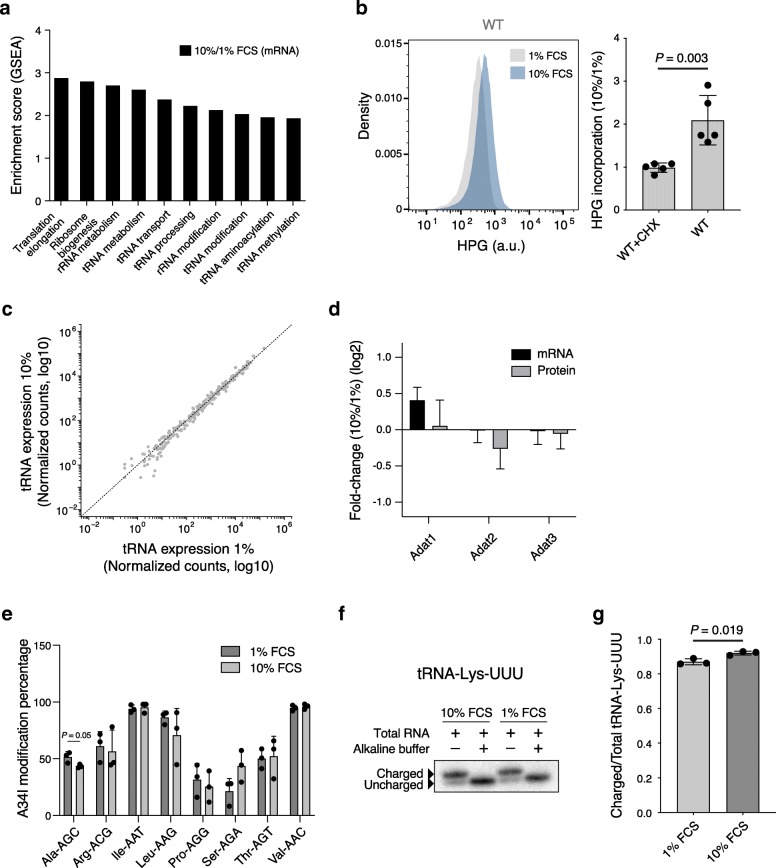
Enhanced translation capacity in proliferating cells. **a** Normalized enrichment score (ES) for translation- and tRNA-related Gene Ontology terms, derived from gene set enrichment analysis (GSEA) comparing gene expression data from cells grown in 10% relative to 1% FCS. For all the pathways depicted, *P* < 0.05. *P* values were calculated by comparing the empirical ES of a gene set to a null distribution of ESs derived from permuting the gene set and then adjusted for multiple hypotheses testing. **b** Representative result of HPG incorporation measurement (a.u., arbitrary units) by flow cytometry in the wild-type NIH-3T3 cells grown in either 1% or 10% FCS (left). Quantification of the mean (± s.e.m.) ratio of HPG incorporation in 10% compared to 1% FCS for the same cells (*n* = 5) (right). For control, we also incubated wild-type NIH-3T3 cells with the translation inhibitor cycloheximide (CHX). Shown are the *P* values determined by the non-parametric Mann-Whitney *U* test. **c** Scatter plot of tRNA gene expression levels (log10, library size-normalized counts) in cells grown in either 10% or 1% FCS. Shown are the mean expression values for each tRNA gene (*n* = 3). The dashed line indicates equal relative abundances in the two conditions. No tRNA genes were significantly upregulated or downregulated between the two conditions (false discovery rate, FDR < 0.01). **d** Mean (± s.e.m.) mRNA and protein fold changes of tRNA deaminases for cells grown in 10% relative to 1% FCS (*n* = 3). **e** Mean (± s.e.m.) percentage of A34I modification in different tRNAs, as inferred from tRNA sequencing data, for cells grown in 10% relative to 1% FCS (*n* = 3). Shown are the *P* values determined by the non-parametric Mann-Whitney *U* test. The amino acid encoded by each codon is shown with a three-letter code: Arg, arginine; Ile, isoleucine; Leu, leucine; Pro, proline; Ser, serine; Thr, threonine; Val, valine. **f** Representative result of charged/uncharged tRNA-Lys-UUU abundances in cells grown in either 10% or 1% FCS using northern blot analysis. Total RNA was also treated with alkaline buffer to deaminoacylate tRNA-Lys-UUU. **g** Mean (± s.e.m.) ratio of charged to total tRNA-Lys-UUU in cells grown in either 10% or 1% FCS using northern blot analysis (*n* = 3). Shown is the *P* value determined by *t* test

To uncover the molecular mechanisms underlying the enhanced translation of mRNAs with rare codons, we performed gene set enrichment analysis (GSEA) using mRNA expression data comparing cells growing in different serum levels. This revealed an upregulation of several translation machinery-related pathways in proliferating cells, including ribosome biogenesis and multiple tRNA metabolism pathways (Fig. 4a). We confirmed that the total protein synthesis rate was significantly increased (~ 2-fold) in cells growing in 10% compared to 1% FCS (Fig. 4b), in agreement with the observations registered for the reporter cell lines growing in similar conditions (Fig. 3g). While increased ribosome concentration could lead to increased translation initiation rates [33], it could not be at the root of observed differences in output between our constructs, which only differ in the codon bias of internal parts of their open reading frames, and thus are most likely affected in their translation elongation rates. Since codon elongation velocity is mostly dependent on the level of cognate tRNAs, we next assessed tRNA pools in cells growing at different proliferation rates by tRNA sequencing [49]. We found no difference in the relative abundance of individual tRNAs between cells growing in 1% and 10% FCS, neither at the gene (Fig. 4c) nor at the isoacceptor level (Additional file 1: Figure S7A), demonstrating that the apparent improvement in elongation efficiency at rare codons did not result from a specific increase in abundance of the cognate tRNAs.

A recent study has proposed that self-renewing embryonic stem cells optimize the translation of codons that depend on the wobble interaction with cognate tRNAs via adenosine-to-inosine modification [26]. Since these codons are a subset of those we found enriched in pro-proliferative transcripts, we investigated the possibility that increased inosine modifications of tRNA underlie the translation reprogramming of proliferative cells. However, we did not observe changes in the abundance of tRNA deaminases (Fig. 4d), and further analysis of tRNA sequencing data showed no increase of inosine modifications in tRNAs (Fig. 4e and Additional file 6: Table S5), as inferred from A-to-G mutations in the anticodon wobble position of tRNAs.

Yet, another model that was proposed to underlie oscillations in translation during the cell cycle of the yeast *Saccharomyces cerevisiae* relies on a global oscillation in the total tRNA pool [39]. Specifically, the expression of a number of tRNA synthetases was found to oscillate during the yeast cell cycle, while the total tRNA concentration was reported to be ~ 2-fold higher in the G2 phase compared to the other cell cycle phases. Such global upregulation was proposed to specifically enhance elongation at codons dependent on the wobble interaction, which are over-represented in genes whose expression oscillates during the cell cycle [39]. Measuring changes in tRNA concentrations is challenging, especially when the effects are small as we expect in our system, where the proportion of cells in G2/M varies only 10–15% between conditions (Fig. 2a). By separating and quantifying the population of RNAs in the tRNA size range, we did not find a significant difference between the cells grown in 1% vs 10% FCS (data not shown). Alternatively, the level of ready-to-translate tRNA can be increased by more efficient charging of these molecules [6]. Since our GSEA analysis suggested changes in tRNA aminoacylation (Fig. 4a), we used a probe for tRNAs decoding the lysine anticodon UUU (tRNA-Lys-UUU) to quantify their charged/uncharged abundance in cells growing in 1% and 10% FCS by northern blotting (Fig. 4f). Starting from the same amount of total RNA, we found that the ratio of charged to total tRNA-Lys-UUU is slightly increased in proliferating cells (Fig. 4g), consistent with an increase in tRNA aminoacylation. Thus, our data indicate that a specific increase in the translation efficiency of mRNAs enriched in rare codons could neither be explained by specific upregulation of the cognate tRNAs nor by increased inosine modification of tRNAs. Instead, our data are most consistent with the model whereby an increase in a general component of the translation elongation machinery upon induction of proliferation preferentially boosts the translation of elongation-limited transcripts carrying rare codons. An analogous mechanism has been suggested to explain how alterations in ribosome concentration may specifically regulate initiation-limited mRNAs [33].

### A general increase in the availability of ready-to-translate tRNAs can selectively improve the decoding of rare codons

**Fig. 5:**
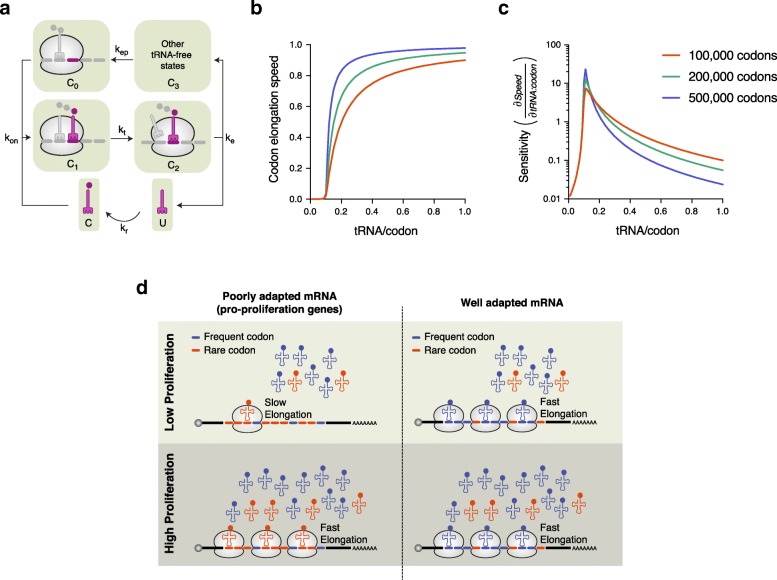
A global increase of ready-to-translate tRNAs can specifically alleviate rare codon-dependent translation bottlenecks in proliferating cells. **a** Schema of one step of the peptide elongation model. For details regarding the different states and rates, please refer to the “Materials and methods” section. Results of simulations showing the dependency of codon elongation speed (**b**) and its sensitivity (**c**) (∂Speed/∂tRNA:codon) to the tRNA:codon ratio, for codons with different abundances in the transcriptome. **d** Conceptual model of the relationship between proliferation rate, codon elongation speed, and the abundance of ready-to-translate tRNAs for mRNAs enriched in rare or common codons. In slowly proliferating cells with limited translation capacity, elongation at rare codons is slow (top left), whereas in rapidly proliferating cells, this translation bottleneck is relieved by a global increase of ready-to-translate tRNAs (bottom left), leading to a faster elongation on pro-proliferation mRNAs containing rare codons. Conversely, mRNAs enriched in common codons will be mostly insensitive to variation in the translation capacity registered in cells with different proliferation rates (top and bottom right)

To find out whether a global change in ready-to-translate tRNA availability could give rise to the transcript and codon-specific effects on elongation speed, we used a mathematical model describing the main biochemical steps involved in ribosome translocation by one codon during the elongation step of translation (Fig. 5a). Assuming a steady state, the speed of elongation for a given codon is:

$$ v=\frac{1}{\tau_{el}}=\frac{k_{on}{k}_tC}{k_t+{k}_{on}C} $$

where *τ*_*el*_ is the time needed for a given codon to be recognized by the cognate tRNA and for translocation to take place, *C* is the number of charged tRNAs, which depends on other parameters (see the “Materials and methods” section), while *k*_*on*_ and *k*_*t*_ are the rates of codon-cognate tRNA binding and ribosome translocation, respectively. Further analysis of this model revealed that for a given tRNA:codon ratio, more abundant codons have higher elongation speed compared to rare codons (Fig. 5b), as expected [11, 12]. However, the speed of elongation shows an ultrasensitive response with respect to the tRNA:codon stoichiometry, exhibiting a peak when the tRNA abundance stops being rate limiting; whereas the height of the peak is greater for common codons, once tRNAs are not limiting, the same increase in the tRNA to codon ratio leads to a higher increase in the elongation rate for rare codons compared to more common codons (Fig. 5c). Similar results were obtained when the sensitivity of decoding speed to changes in the tRNA charging rate (*k*_*r*_) was determined. Specifically, when *k*_*r*_ increases, the elongation speed of rare codons increases more than that of common codons. Thus, while the precise parameter that changes as cells are induced to proliferate needs to be identified, our results are most consistent with an overall increase in tRNA availability leading to a stronger boost in the translation of transcripts that are enriched in rare codons compared to other transcripts. This would be a reflection of translation elongation being most limited for rare codon-enriched transcripts in cells when the overall elongation machinery is reduced, such as cells growing at low proliferation rates [33, 34, 39, 50, 51].

## Discussion

Our results reveal an unexpected translation program encoded in proliferation-related mRNAs, which are enriched in rare codons. Although rare codons are generally associated with reduced translation output [9, 11, 12], we here demonstrate that in proliferating cells, these codons enable a higher boost in translation efficiency compared to common codons (Fig. 5d). This suggests that induction of proliferation is associated with the removal of a translation bottleneck, consistent with reports of increased availability of various components of the protein synthesis apparatus in these conditions [39, 52]. The nature of the translation bottleneck remains to be further defined. Here, we explore a few scenarios, previously proposed to explain how tRNAs can drive codon-mediated changes in translation. We do not find tRNA genes to be individually regulated in cells proliferating at different rates, in agreement with a few reports [29, 39], but not with an initial study comparing the proliferation/differentiation state of cells [24]. Profiling of tRNA expression being very challenging [49, 53], it remains possible that more accurate methods can reveal some differences in the expression of individual tRNAs between cellular states. However, the coverage of tRNAs by reads in our sequencing dataset, though not uniform over the tRNA length, was relatively similar for tRNAs decoding codons with A/U or G/C at the third nucleotide position (Additional file 1: Figure S7B), making it unlikely that a sequence-dependent bias in the sample preparation has masked true changes in individual tRNA abundance between conditions.

The model that remains most consistent with our data is that global changes in translation capacity differentially affect the translation output of transcripts with pro-proliferative functions compared to other mRNAs, due to distinct codon biases. Pro-proliferative mRNAs enriched in rare codons are elongation-limited when cells are growing slowly, as cognate tRNAs are present in low abundance. If the availability of ready-to-translate tRNAs increases globally upon induction of proliferation, transcripts enriched in rare codons will cease to be elongation-limited and will exhibit a preferential boost in protein synthesis rates [46, 54]. Both reduced ribosome collisions and reduced drop-off events could contribute to this effect. Improved elongation can also lead to higher transcript initiation rates by faster ribosome clearance within the initiation region or by increasing ribosome recycling, both of which would result in a further overall increase in protein synthesis rates. It is clear that this model needs further experimental validation, currently hampered by a lack of methods for systematically quantifying global tRNA pools in specific and possibly small populations of cells. Irrespective of this, our study emphasizes the nuanced and dynamic nature of translation and the role of rare codons in this process. Transcripts carrying rare codons, which are distinct across tissues and implement different functions (Additional file 1: Figure S2), are not simply hampered in translation compared to transcripts encoded with common codons, but undergo a stronger boost in translation in specific conditions, when the availability of translation elongation machinery increases globally.

## Conclusion

Our study shows that rare codons may serve a dual purpose: (1) to stabilize the resting state of cells by limiting the expression of pro-proliferative genes when tRNA pools are limited and (2) to specifically boost the protein output of pro-proliferative relative to other mRNAs, when cells need to divide rapidly. We provide direct evidence that codon bias is exploited to yield dynamic changes in translation efficiency and that rare codons, rather than persisting passively in genomes due to reduced selection for translation efficiency, may be actively used to modulate translation rates. Our data indicate that induction of proliferation leads to the removal of a translation bottleneck that affects transcripts enriched in rare codons. Of the tRNA-related models proposed so far to underlie translational changes, our data are most consistent with a global change in the tRNA pool differentially affecting translation of transcripts with distinct codon biases.

## Materials and methods

### Cell culture

All NIH-3T3 cell lines were cultured and maintained in DMEM (SIGMA-ALDRICH, Cat# D5671) supplemented with 2 mM glutamine (GIBCO, Cat# 25030-024), 100 U penicillin/0.1 mg/ml streptomycin (Sigma, Cat# P4333), and 10% heat-inactivated fetal calf serum (FCS) (BioConcept, Amimed, Cat# 2-01F30-l). For the experiments comparing resting and proliferating cells, we seeded and cultured the cells either with 1%, 2%, 5%, or 10% FCS for 48 h.

### Development of a FUCCI cell line and sorting of cells in different cell cycle phases

NIH-3T3 cells (ATCC, Cat# CRL-1658), cultured normally, were used to create a stable FUCCI cell line [40]. The following vectors were used to generate the FUCCI system: (1) pRetroX-G1-Red (Clontech, Cat# 631463) and (2) pRetroX-SG2M-Cyan (Clontech, Cat# 631462). For particle formation and replication, the plasmids were transfected into the packaging Phoenix-AMPHO cell line (ATCC, Cat# CRL-3213) according to the manufacturer’s instructions. To make a stable cell line, the parental cells were first transduced with 10 ml of viral supernatant containing the pRetroX-G1-Red vector mixed with 8 μg/ml polybrene (SIGMA-ALDRICH, Cat# TR-1003) for 8 h. After 48 h, cells were passaged and cultured in standard medium containing 0.8 μg/ml of the selection marker puromycin. The cells were then propagated to 10 15-cm plates and grown to approximately 50% confluence, after which they were harvested with Accutase (Thermo Fisher Scientific, Cat# A1110501) and a uniform single-cell suspension was generated by filtering the harvest through 30-μM Miltenyi filters (Miltenyi Biotec Cat# 130-041-407) and suspended in 2 ml of FACS buffer (PBS with 2% FBS). The cells were then sorted on a BC MoFlo XDP cell sorter (Beckman Coulter) for positive mCherry expression using the 561 laser, BP Filter 615/20. Non-transduced NIH-3T3 cells, not expressing mCherry, were used as control. The sorted cells were put back in the culture and were transduced analogously with the pRetroX-SG2M-Cyan vector. After 48 h, cells were cultured in standard medium containing both 0.8 μg/ml of the selection marker puromycin and 0.8 μg/ml of the selection marker geneticin (Thermo Fisher Scientific, Cat# 10131027) and passaged for 2 weeks to allow for proper selection of transduced cells. The cells were again propagated to 10 15-cm plates and grown to approximately 50% confluence, after which they were harvested with Accutase (Thermo Fisher Scientific, Cat# A1110501) and a uniform single-cell suspension was generated by filtering the harvest through Miltenyi filters and suspended in 2 ml of FACS buffer (PBS with 2% FBS). Transduced cells were then sorted on a BC MoFlo XDP cell sorter (Beckman Coulter) for positive amCyan expression using the 445 laser, BP Filter 510/20. Non-transduced NIH-3T3 cells, expressing neither mCherry nor amCyan, were used as control. Finally, cells were put back in the culture in standard medium containing both selection markers.

The stable cell line with the integrated FUCCI system was then used to collect cells in different cell cycle phases. In every FACS experiment, 15 15-cm plates with FUCCI cells were grown, harvested, prepared, and sorted as described above. Immediately following the sorting, cells were centrifuged, and pellets were snap-frozen. These pellets were then used for the various mRNA library preparations.

### mRNA sequencing of cells in different cell cycle phases

Libraries were prepared for mRNA sequencing using “Directional mRNA-seq sample preparation” protocol from Illumina with minor modifications. In brief, poly(A)+ RNA was isolated directly from cells using Dynabeads® mRNA DIRECT™ Kit (Life Technologies, Cat# 61011) according to the manufacturer’s protocol. After isolation, 50 ng of mRNA was chemically fragmented by incubating mRNA solution with twice the volume of alkaline hydrolysis buffer (50 mM sodium carbonate [NaHCO3/Na2Co3] pH 9.2, 1 mM EDTA) at 95 °C for 5 min to get the fragments of approximately 200–300 bases. Fragmented mRNA was immediately purified with RNeasy MinElute Cleanup Kit (Qiagen, Cat# 74204) to stop the reaction and to remove small RNA fragments (< 100 bases). Further, purified fragmented mRNA was treated with thermo-sensitive alkaline phosphatase FastAP (Fermentas, Cat# EF0651) at 37 °C for 30 min and then at 75 °C for 5 min to inactivate FastAP. Fragmented mRNA was further incubated with ATP and T4 polynucleotide kinase (Fermentas, Cat# EK0032) at 37 °C for an hour and subsequently purified. Ligation of RNA 3′ adapter (Illumina, RA3, Part# 15013207) was done using T4 RNA Ligase 2, truncated K227Q (New England Biolabs Inc., Cat# M0351 L) according to the Illumina protocol. The ligation step is followed by RNA purification as mentioned above to remove unligated 3′ adapters. The RNA 5′ Adapter (Illumina, RA5, Part# 15013205) was ligated using T4 RNA ligase (Fermentas, Cat# EL0021) according to the Illumina protocol followed by RNA purification step to remove unligated 5′ adapters. cDNA was synthesized using RNA RT Primer (Illumina, RTP, Part# 15013981) using SuperScript III (Invitrogen, Cat# 18080044) as per the Illumina protocol. Libraries were amplified for 14 cycles of PCR using forward PCR primer (Illumina, RNA PCR Primer (RP1), Part# 15005505) and reverse PCR primer (Illumina PCR Primer, Index). Different indexed reverse PCR primers were used for library preparation from different samples to facilitate multiplexing. Libraries were sequenced for 51 cycles on Illumina HiSeq 2000 machine.

### Generation of stable cell lines expressing different GFP reporters

The two re-coded fluorescent reporters were based on the d2eGFP protein [48], which is the enhanced green fluorescent protein (eGFP) with a C-terminal extension consisting in amino acids 422–461 of the degradation domain of mouse ornithine decarboxylase (PEST sequence). The rapid turnover rate of this reporter protein makes it ideal for observing dynamic changes in protein synthesis [48]. To design the two re-coded fluorescent reporters, we have altered each codon of the d2eGFP coding sequence to match the codon bias of either G1- or G2M-enriched genes, yielding two reporters: d2eGFP_G1 and d2eGFP_G2M, respectively (Additional file 7: Table S6). The first and last 17 codons (51 nucleotides) of eGFP, as well as the PEST sequence, were left unchanged to avoid potential effects on the translation initiation and termination of the reporter transcripts. In silico-designed sequences were first synthesized into double-stranded DNA fragments by IDT (Skokie, IL 60076, USA) using gBlocks technology and, then, cloned into the pCDH-EF1-MCS-T2A-Puro vector (System Biosciences, Cat# CD527A-1). To generate a stable cell line, we grow the HEK-293T cell line in a 6-well plate and produce the pseudo-virus using the Lenti-vpak Lentiviral Packaging Kit (Origene, Cat# TR30022) according to the manufacturer’s instructions. After 48 h, the media containing pseudo-viruses were collected, filtered through 0.45-μm syringe filter (Sarstedt, Cat# 83.1826). For transduction, we added the pseudo-viral supernatant containing 8 μg/ml polybrene to NIH-3T3 cells growing in a 6-well plate. For the selection of transduced cells, we changed the media after 48 h with normal media containing 1.0 μg/ml of puromycin. Finally, the 2 stable cell lines expressing d2eGFP_G1 and d2eGFP_G2M were sorted on a BD FACSAria III (Beckman Coulter) using the 488 laser, BP filter 514/30 so as to display a similar level of green fluorescence. We also used an empty pCDH-EF1-MCS-T2A-Puro vector to generate a stable control cell line (pCDH-empty) using the protocol mentioned above.

### Quantification of GFP expression and cell cycle phase for the different reporters

Cells were grown in either 1% or 10% FCS as described in the “Cell culture” section. For this assay, we seeded 8 × 10^5^ cells in 10-cm plates for the pCDH-empty, d2eGFP_G1, and d2eGFP_G2M cell lines. After 48 h, we performed a flow cytometry analysis using 0.6 × 10^6^ cells resuspended in 1 ml of corresponding media and stained with Hoechst 33342 (SIGMA ALDRICH, Cat# B2261) at a final concentration of 15 μg/ml for 60 min at 37 °C with gentle shaking to avoid settling of the cells. After incubation, 4 ml of PBS was added and the samples were centrifuged at 1000 RPM for 3 min at room temperature. The cell pellet was resuspended in 300 μl of HBSS (Gibco, Cat# 14025-050) with 2% FCS and subsequently used for flow cytometry analysis on a BD LSR Fortessa (Beckman Coulter) using the 405 laser, BP filter 450/50 (for Hoechst measurement) and the 488 laser, BP filter 512/25 (for GFP measurement).

For each biological replicate of the two reporter cell lines, GFP fluorescence intensity, as well as the fraction of cells in G1 and G2/M phases (based on Hoechst staining), was estimated by averaging the measurements from two technical replicates. Then, we calculated the ratio of GFP fluorescence intensity in cells grown in 10% relative to 1% FCS.

### Transcriptomic profiling with mRNA-seq for cells grown in different serum levels

Cells were grown in either 1% or 10% FCS as described in the “Cell culture” section. For this experiment, we seeded 8 × 10^5^ cells in 10-cm plates for the pCDH-empty cell line. After 48 h, total RNA was isolated using Direct-zol RNA MiniPrep kit (Zymo Research, Cat #R2050) according to the manufacturer’s manual. Total RNA was quality-checked on the Bioanalyzer instrument (Agilent Technologies, Santa Clara, CA, USA) using the RNA 6000 Nano Chip (Agilent, Cat# 5067-1511) and quantified by spectrophotometry using the NanoDrop ND-1000 Instrument (NanoDrop Technologies, Wilmington, DE, USA). One microgram total RNA was used for library preparation with the TruSeq Stranded mRNA Library Prep Kit High Throughput (Illumina, Cat# RS-122-2103). Libraries were quality-checked on the Fragment Analyzer (Advanced Analytical, Ames, IA, USA) using the Standard Sensitivity NGS Fragment Analysis Kit (Advanced Analytical, Cat# DNF-473). The samples were pooled to equal molarity. Each pool was quantified by PicoGreen Fluorometric measurement to be adjusted to 1.8 pM and used for clustering on the NextSeq 500 instrument (Illumina). The samples were sequenced using the NextSeq 500 High Output Kit 75-cycles (Illumina, Cat# FC-404-1005). Primary data analysis was performed with the Illumina RTA version 2.4.11 and base calling software version bcl2fastq-2.20.0.422.

### mRNA-seq differential expression analysis

mRNA-seq reads were subjected to 3′ adapter trimming (Additional file 7: Table S6) and quality control (reads shorter than 20 nucleotides or for which over 10% of the nucleotides had a PHRED quality score < 20, were discarded). Filtered reads were then mapped to the mouse transcriptome based on genome assembly mm10 and transcript annotations from RefSeq (November 2017) with segemehl v0.1.7-411 [55] allowing a minimum mapping accuracy of 90%. Transcript counts were calculated based on uniquely mapped reads and used for differential expression analysis with DESeq2 [56]. Upregulation and downregulation of mRNAs were considered significant when the false discovery rate was lower than 0.01.

Two biological replicates were obtained for each cell cycle phase using the FUCCI system. Library sizes (before quality control) ranged from 20 to 35 million reads, and mapping rates varied between 71 and 75% across replicates. The large number of uniquely mapped reads to mRNAs (~ 12–20 million) allowed a robust quantification of genome-wide transcript abundances (Spearman correlations between biological replicates of 0.972–0.973).

Four biological replicates were obtained for each serum condition (1% or 10% FCS) for the pCDH-empty cell line. Library sizes (before quality control) ranged from 30 to 40 million reads, and mapping rates varied between 78 and 82% across replicates. The large number of uniquely mapped reads to mRNAs (~ 16–20 million) allowed a robust quantification of genome-wide transcript abundances (Spearman correlations between replicates of 0.975–0.978).

### Ribosome occupancy profiling for cells grown in different serum levels

Ribosome occupancy profiling protocol was adapted from [57]. In brief, cells were grown in either 1% or 10% FCS as described in the “Cell culture” section. For each library preparation, we seeded 5 × 10^6^ cells in 15-cm plates for cells grown in 1% (two plates) and 10% (one plate) FCS, respectively. After 48 h, the cells were washed twice with ice-cold PBS containing 100 μg/ml cycloheximide (Sigma, Cat# C1988-1G) on ice. The cells were extensively scraped in presence of 500 μl polysome lysis buffer (20 mM Tris-HCl, 100 mM NaCl, 10 mM MgCl_2_, 1% Triton X-100, 2 mM dithiothreitol, 100 μg/ml cycloheximide, 500 U/ml RNasin plus, protease inhibitor mini Complete EDTA free), collected, triturated with the pipette, and incubated for 10 min on ice. Further, the lysate was clarified by passing through a 26-G needle for five to seven times and followed by centrifugation at 20,000*g* for 10 min at 4 °C. The clarified lysate was snap frozen and stored at − 80 °C if required.

10–50% linear sucrose gradient was prepared in gradient buffer (50 mM Tris-HCl (pH = 7.5), 50 mM NH_4_Cl, 12 mM MgCl_2_, 0.5 mM DTT, 100 μg/ml CHX) using Gradient Master instrument (Biocomp) according to the instrument instruction and precooled for 1 h at 4 °C. In parallel, a fraction of lysate equivalent to *A*_260_ = 10 was treated with 8 μl of 1:10 diluted RNase I (100 U/μl, Ambion, Cat# AM2295) for 45 min at 22 °C with gentle agitation, and RNase I was inactivated by addition of 10 μl of SUPERaseIn (20 U/μl, Ambion, Cat# AM2696). The samples were immediately loaded on the precooled linear gradient and centrifuged at 35000 rpm for 3 h at 4 °C in a TH-641 rotor (Thermo Scientific). For ribosome profiling, different fractions of the gradient were collected in 1% SDS solution using Density Gradient Fractionation System (Brandel) with a setting of pump speed (0.75 ml/min), detector sensitivity at 1.0, and collection time 32 s per tube and flash frozen.

The appropriate fractions that contain monosomes were processed for footprint library preparation according to [58]. In brief, RNA was isolated from the collected monosomes fraction with the phenol-chloroform method. RNA fragments of appropriate size (28–30 nt) were selected by running samples on 15% polyacrylamide denaturing TBE-Urea gel and visualized by SYBR Gold (Life Technologies, Cat# S11494). Size-selected RNA was dephosphorylated by T4 polynucleotide kinase (PNK, New England Biolabs, Cat# M0201 L) treatment for 1 h at 37 °C. PNK was heat inactivated, and RNA was purified using the phenol-chloroform method and overnight precipitation of RNA in ethanol at − 20 °C. Preadenylated 3′ linker was ligated to dephosphorylated RNA by using T4 RNA ligase 2, truncated (New England Biolabs, Cat# M0242 L). The ligation reaction was carried out for 4 h at 22 °C. The ligation reaction was run on 15% polyacrylamide denaturing TBE-Urea gel to separate and purify the ligated product from the unligated product and unused 3′ linker. Gel-purified ligated RNA was reverse transcribed by Superscript III (Invitrogen) for 30 min at 48 °C in a total reaction volume of 20 μl. After reverse transcription, RNA was hydrolyzed by adding 2.2 μl of 1 N NaOH solution and incubating for 20 min at 98 °C. First-strand cDNA was further gel purified by electrophoresis on 12% polyacrylamide denaturing TBE-Urea gel and circularized by incubating with CircLigase™ II ssDNA Ligase (Epicentre, Cat# CL9025K) for 60 min at 60 °C followed by inactivation of CircLigase by heating at 80 °C for 10 min. cDNA was subjected to rRNA depletion by subtractive hybridization as described in [57]. Thereafter, circular cDNA was PCR amplified, and then the amplified products were gel purified on 8% native polyacrylamide gel. The prepared library was sequenced on the Illumina Nextseq platform.

### Differential expression analysis of ribosome protected fragments

Ribosome protected fragment (RPF) reads were subjected to 3′ adapter trimming (Additional file 7: Table S6) and quality control (reads shorter than 20 nucleotides or for which over 10% of the nucleotides had a PHRED quality score < 20 were discarded). Filtered reads were then mapped to the mouse transcriptome based on genome assembly mm10 and transcript annotations from RefSeq (November 2017) with segemehl v0.1.7-411 [55] allowing a minimum mapping accuracy of 90%. Transcript counts were calculated based on uniquely mapped reads to the coding sequence and used for differential expression analysis with DESeq2 [56].

Three biological replicates were obtained for each serum condition (1% or 10% FCS). Library sizes (before quality control) ranged from 60 to 80 million reads, and mapping rates varied between 83 and 90% across replicates. Despite an unusually high abundance of rRNA species (~ 80–92% of total mapped reads), the number of uniquely mapped reads to mRNAs (~ 4–9 million) still allowed a robust quantification of genome-wide ribosome occupancies (Spearman correlations between replicates of 0.981–0.988).

The sequenced reads had the expected length (28–34 nt), and for each read length, the relative location of the A-site with respect to the read start was inferred as the offset value for which the 3-nt periodicity was most apparent (the number of reads at the first frame being larger than at both other frames). Only read lengths showing the expected 3-nt periodicity along the CDS were considered for further analyses requiring a codon-level resolution.

The fold changes for ribosome density were calculated by dividing the RPF fold change by the mRNA fold change (RPF_fold change_/mRNA_fold change_). Genes whose ribosome density changed more than 50% in either direction between the two conditions were considered upregulated/downregulated.

### Proteomics and transcriptomics profiling for cells grown with different serum concentrations

Cells were grown in either 1%, 2%, 5%, or 10% FCS as described in the “Cell culture” section. For this experiment, we seeded 8 × 10^5^ cells in 10-cm plates from the parental cell line. Transcriptomic profiling was performed as described above in the “Transcriptomic profiling with mRNA-seq for cells grown in different serum levels” section. Differential expression analysis for mRNA-seq data was performed as described above in the “mRNA-seq differential expression analysis” section. Three biological replicates were obtained for each serum condition (1%, 2%, 5%, or 10% FCS) for the parental cell line. mRNA sequencing library sizes (before quality control) ranged from 20 to 30 million reads, and mapping rates varied between 74 and 86% across replicates. The large number of uniquely mapped reads to mRNAs (~ 14–20 million) allowed a robust quantification of genome-wide transcript abundances (Spearman correlations between replicates of 0.9696–0.978).

### Proteomics sample preparation

Cell pellets (obtained from one well of a 6-well plate) were lysed in 50 μl of lysis buffer (1% sodium deoxycholate, 10 mM TCEP, 100 mM Tris, pH = 8.5) using 10 cycles of sonication (30 s on, 30 s off, Bioruptor, Diagenode). Protein concentration was determined by Reducing Agent Compatible BCA assay (Thermo Fisher Scientific). Sample aliquots containing 100 μg of total proteins were reduced for 10 min at 95 °C and alkylated at 15 mM chloroacetamide for 30 min at 37 °C. Proteins were digested by incubation with sequencing-grade modified trypsin (1/50 w/w; Promega, Madison, WI) overnight at 37 °C. After digestion, the samples were acidified with TFA to a final concentration of 1%. Peptides were cleaned up using PreOmics Cartridges (PreOmics, Martinsried, Germany) following the manufacturer’s instructions. After drying the samples under vacuum, the peptides were resuspended in 0.1% aqueous formic acid solution at a concentration of 0.5 mg/ml.

### LC-MS/MS data acquisition

For each sample, aliquots of 0.5 μg of peptides were analyzed by LC-MS. A chromatic separation was carried out using an EASY nano-LC 1200 system (Thermo Fisher Scientific), equipped with a heated RP-HPLC column (75 μm × 37 cm) packed in-house with 1.9 μm C18 resin (Reprosil-AQ Pur, Dr. Maisch). Peptides were separated using a stepwise gradient ranging from 95% solvent A (0.15% formic acid, 2% acetonitrile) and 5% solvent B (80% acetonitrile, 20% water, 0.15% formic acid) to 50% solvent B over 90 min at a flow rate of 200 nl/min (5–12% B by 5 min, 12–35% B by 65 min and 35–50% B by 90 min). Mass spectrometry analysis was performed on Orbitrap Fusion Lumos mass spectrometer equipped with a nanoelectrospray ion source (both Thermo Fisher Scientific). Each MS1 scan was followed by high-collision dissociation (HCD) of the 20 most abundant precursor ions with a dynamic exclusion for 30 s. For MS1, 1e6 ions were accumulated in the Orbitrap cell over a maximum time of 50 ms and scanned at a resolution of 240,000 FWHM (at 200 m/z). MS2 scans were acquired in the linear ion trap at a target setting of 1e4 ions, with an accumulation time of 35 ms. Singly charged ions and ions with unassigned charge state were excluded from triggering MS2 events. The normalized collision energy was set to 35%, the mass isolation window was set to 1.4 m/z, and one microscan was acquired for each spectrum.

### Label-free quantification

The acquired raw files were imported into the Progenesis QI software (v2.0, Nonlinear Dynamics Limited), which was used to extract peptide precursor ion intensities across all samples applying the default parameters. The generated mgf files were searched using the MASCOT algorithm (Matrix Science, version 2.4.1); the mgf files were searched against a database containing normal and reverse sequences of UniProt entries of *Mus musculus* (March 07, 2019) and commonly observed contaminants (in total 34,794 sequences. The search criteria were set as follows: full tryptic specificity was required (cleavage after lysine or arginine residues, unless followed by proline), 3 missed cleavages were allowed, carbamidomethylation (C) was set as fixed modification, oxidation (M) and N-terminal acetylation were set as variable modifications, and mass tolerance of 10 ppm (precursor) and 0.6 Da (fragments). The database search results were imported into Progenesis QI software, and the list with quantified peptides was exported. The quantitative data were further processed and statistically analyzed using the SafeQuant software tool [59]. In brief, the false discovery rate (FDR) of peptide and protein identification was set to 1%. For quantification, the analysis included global data normalization by equalizing the total peak areas across all LC-MS runs and summation of peak areas per protein. The summarized protein expression values were used for statistical analysis using Bayes moderated *t*-statistics. Finally, the calculated *P* values were corrected for multiple testing using the Benjamini-Hochberg method.

### Quantification of eIF2α and p-eIF2α for cells grown in different serum concentrations

Cells were grown in either 1% or 10% FCS as described in the “Cell culture” section. The western blot protocol was adapted from our previous work [58]. Cells were lysed in 300 μl RIPA buffer containing protease inhibitor and phosphatase inhibitor. Fifteen to 25 μg total protein was loaded on 10% SDS PAGE, and electrophoresis was performed for 1 h at 120 V to resolve the proteins. We followed the protocol from Cell Signaling for transferring, blocking, incubating, washing, and developing the membrane. Cells treated with 1 μM thapsigargin (TG) and 1 μM hydrogen peroxide for 5 h were also included as a positive control for phosphorylation of eIf2α protein. Tubulin was used as a loading control. The following antibodies were used in the western blot analysis: Phospho-eIF2α (Ser51) Antibody (Cell Signaling #9721), eIF2 α Antibody (Cell Signaling #9722), and α-Tubulin Antibody (Merck Millipore #CP06).

For quantification, band intensities of eIF2α and p-eIF2α were normalized by the corresponding loading control (tubulin). Then, for both eIF2α and p-eIF2α, normalized band intensities were divided by the average normalized band intensity across the different conditions. Finally, the ratio of p-eIF2α to eIF2α was calculated by dividing the normalized intensities calculated in the previous step. The band intensities were quantified using the ImageJ software [60].

### Quantification of aminoacyl tRNA levels by northern blot

Cells were grown in either 1% or 10% FCS as described in the “Cell culture” section. The northern blot protocol was adapted from [61] with minor modifications. In brief, total RNA was isolated using TRI Reagent® (Sigma-Aldrich #T9424) as described in the manufacturer’s protocol. After the final step of 75% ethanol wash, the RNA pellet was resuspended in acidic RNA storage buffer (Sodium acetate 10 mM; pH = 5.2, EDTA 1 mM) to preserve the aminoacyl state of tRNAs. Five micrograms of total RNA was loaded onto 8% acidic polyacrylamide Urea gel containing 8 M urea, 100 mM sodium acetate, 1 mM EDTA. An equal amount of RNA from each sample was alkali treated by adding an equal volume of alkaline buffer (10 mM Tris-HCl; pH 8.9, 1 mM EDTA) at 72 °C for half an hour and included in gel electrophoresis to determine the gel migration position of deacetylated tRNA. The electrophoresis was performed in 100 mM sodium acetate buffer (pH ~ 5.2) for 36 h at 60 V at 4 °C. Following electrophoresis, RNA was transferred to a positively charged nylon membrane and subsequently crosslinked to the membrane using Strata UV crosslinker for 2 min. The membrane was pre-hybridized for 30 min at 42 °C with ULTRAhyb® Ultrasensitive Hybridization Buffer (Thermo Fisher Scientific #AM8669). Thereafter, 20 pmol of ^32^P-labeled ssDNA probe against UUU-tRNA (5′-GCCCGGATAGCTCAGTCGGTAGAGCATCAGACTTTTAATCTGAGGGTCCAGGGTTCAAGTCCCTGTTCGGGCG-3′) was added to the hybridization buffer and incubated for 16 h at 42 °C. After hybridization, the membrane was washed according to the manufacturer’s protocol for the hybridization buffer. Additionally, the membrane was finally washed with 0.1% SDS buffer for overnight at 42 °C. For detection, the membrane was exposed to phosphorimager screen for 8 h, and the image was captured on a phosphorimager.

The ratio of charged to total tRNA was quantified by dividing the intensity of the band corresponding to charged tRNA by the sum of intensities for the bands of charged and uncharged tRNA. The band intensities were quantified using the ImageJ software [60].

### Quantitative RT-PCR analysis

Cells were grown in either 1% or 10% FCS as described in the “Cell culture” section. For this experiment, we seeded 8 × 10^5^ cells in 10-cm plates for the pCDH-empty, d2eGFP_G1, and d2eGFP_G2M cell lines. After 48 h, total RNA was isolated using the Direct-zol RNA MiniPrep kit (Zymo Research, Cat #R2050) according to the manufacturer’s manual. We then synthesized cDNA according to the protocol described in Super Script III First Strand Synthesis System for qRT-PCR kit (Invitrogen, Cat# 18080-044). After cDNA synthesis, the RNA was digested using RNAse H (NEB, Cat# M0297 L), and the reaction was purified using Qiagen PCR purification kit (Qiagen, Cat# 28104).

For quantitative PCR analysis, we used the GoTaq qPCR Master mix kit (Promega, Cat# A6002). For qPCR reaction setup, we used 1 μl of cDNA, 0.2 μM of primers (Additional file 7: Table S6), 10 μl of GoTaq qRT-PCR master mix (2×), and water to make a final volume of 20 μl per reaction. The reaction was run on OneStepPlus qPCR machine from Applied Biosciences. The amplification program was as follows: initial denaturation at 95 °C for 10 min, 40 cycles at 95 °C for 30 s, and 60 °C for 1 min. After amplification, a thermal denaturing cycle was added to derive the dissociation curve of the PCR product to verify amplification specificity. Reactions for each sample were carried out in triplicate.

For each biological replicate, d2eGFP_G1 and d2eGFP_G2M transcript abundances were estimated relative to the endogenous *Gapdh* gene after averaging the measurements from three technical replicates. Relative fold changes in the gene expression between 1 and 10% FCS were calculated using the comparative 2^−ΔΔCt^ method [62].

### Quantification of nascent protein synthesis

To quantify nascent protein synthesis as a measure of global translation, we used the non-radioactive metabolic labeling assay Click-iT HPG Alexa Fluor 594 Protein Synthesis Assay Kit (Thermo Fisher Scientific, Cat #C10429). The method is based on the incorporation of L-HPG, an amino acid analog of methionine containing an alkyne moiety, and Alexa Fluor 594 azide. For this assay, we seeded 7 × 10^5^ cells per well in 6-well plates for the parental, d2eGFP_G1, and d2eGFP_G2M cell lines and let them grow for 24 h. We changed the media with a l-methionine-free medium DMEM (Gibco, Cat#21013) supplemented with 2 mM glutamine, 100 U penicillin/0.1 mg/ml streptomycin, and 10% heat-inactivated FCS and 50 μM HPG, and incubated for 3 h at 37 °C and 7% CO_2_. For the control, we also incubated parental cells with an additional 35 μg/ml of cycloheximide. After incubation, cells were harvested with Accutase (Thermo Fisher Scientific, Cat# A1110501) and further processed according to the kit protocol. The signal intensity of incorporated HPG-Alexa Fluor 594 was measured by flow cytometry on a BD LSR Fortessa (Beckman Coulter) using the 561 laser, BP filter 610/20 laser. Mean fluorescence intensities were computed from 50,000 cells for each sample. Fluorescence intensity for each cell line growing in 10% FCS was normalized by the mean fluorescence intensity of the same cell line growing in 1% FCS. The experiment was performed with five biological replicates.

### tRNA abundance profiling with tRNA-seq for cells grown in different serum levels

Cells were grown in either 1% or 10% FCS as described in the “Cell culture” section. For this experiment, we seeded 8 × 10^5^ cells in 10-cm plates for the pCDH-empty, d2eGFP_G1, and d2eGFP_G2M cell lines. After 48 h, total RNA was isolated using Direct-zol RNA MiniPrep kit (Zymo Research, Cat# R2050) according to the manufacturer’s manual. The tRNA-seq method was performed by Arraystar Inc. (Rockville, MD 20850, USA). Briefly, total RNA from each sample was quantified using a NanoDrop ND-1000 instrument. tRNAs were purified from total RNA and partially hydrolyzed according to the Hydro-tRNAseq method [49]. Then, partially hydrolyzed and re-phosphorylated tRNAs were used to prepare the sequencing library in the following steps: (1) 3′-adapter ligation, (2) 5′-adapter ligation, (3) cDNA synthesis, (4) PCR amplification, and (5) size selection of ~ 140–155 bp PCR-amplified fragments (corresponding to ~ 19–35 nt tRNA fragments size range). The libraries were denatured as single-stranded DNA molecules, captured on Illumina flow cells, amplified in situ as sequencing clusters, and sequenced for 75 cycles on Illumina NextSeq system as per the manufacturer’s instructions.

### tRNA-seq differential expression analysis

tRNA-seq reads were subjected to 3′ adapter trimming (Additional file 7: Table S6) and quality control (reads shorter than 20 nucleotides or for which over 10% of the nucleotides had a PHRED quality score < 20 were discarded). Filtered reads were then mapped to an artificial mouse transcriptome based on genome assembly mm10 and annotations for rRNA and tRNA genes from RefSeq and GtRNAdb (http://gtrnadb.ucsc.edu, January 2018) [63], respectively, with segemehl v0.1.7-411 [55] allowing a minimum mapping accuracy of 90%. tRNA gene counts were calculated based on uniquely and multi-mapped reads, where the latter were evenly split across all compatible tRNA genes, and used for differential expression analysis with DESeq2 [56]. Upregulated and downregulated genes were considered significant if the corresponding false discovery rate was lower than 0.01.

Three biological replicates were obtained for each serum condition (1% or 10% FCS). Library sizes (before quality control) ranged from 10 to 12 million reads, and mapping rates varied between 64 and 82% across replicates. The large number of mapped reads to tRNAs allowed a robust quantification of their abundances (Spearman correlations between replicates of 0.970–0.992).

### Quantification of inosine levels in tRNAs using tRNA-seq

Inosine at the first residue of the anticodon will form the strongest bond to cytosine and it will therefore appear as a guanine following sequencing. Thus, the level of A34I modifications was quantified by measuring the A-to-G substitutions at the anticodon wobble position using samtools mpileup v1.9 (http://samtools.sourceforge.net) [64] and VarScan v2.4.1 (http://varscan.sourceforge.net) [65]: samtools mpileup -B -f reference.fa sample.sam | java -jar VarScan.v2.4.1.jar mpileup2snp --min-coverage 10 --min-var-freq 0 --p-value 0.05 --variants --output-vcf --strand-filter 0

### Prediction of RNA structure in the translation initiation region

Minimum free energy (MFE) of folding of the 80 nucleotide region centered on the start codon of mouse genes was calculated using UNAFold v3.8 (http://unafold.rna.albany.edu/) [66] with default parameters.

### Calculation of codon usage

The frequency of each codon in every protein-coding mouse transcript, based on genome assembly mm10 and transcript annotations from RefSeq (November 2017), was calculated using codonR vFeb2005 (http://people.cryst.bbk.ac.uk/~fdosr01/tAI/). Then, for each transcript, the relative usage of different synonymous codons was calculated by dividing the frequency of usage of each codon by the total frequency of codons encoding the same amino acid.

### Determination of tissue-specific transcripts

mRNA-seq data for different mouse tissues was retrieved from [67] (GSE29184). Kallisto v0.43.0 (https://pachterlab.github.io/kallisto/) [68] (parameters: --single -l 200 and -s 20) was used to map the reads to a reference transcriptome based on genome assembly mm10 and transcript annotations from RefSeq (November 2017). Transcript counts were then used for differential expression analysis with DESeq2 [56]. Transcripts were defined as tissue-specific if their expression in that particular tissue compared to all other tissues was greater than twofold and the corresponding false discovery rate was lower than 0.01.

### Calculation of the tRNA adaptation index

tRNA adaptation index (tAI) [69] for each protein-coding transcript was calculated using codonR vFeb2005 (http://people.cryst.bbk.ac.uk/~fdosr01/tAI/). Mouse transcriptome was based on genome assembly mm10 and transcript annotations from RefSeq (November 2017). In brief, to calculate the tAI for a gene, we first needed to calculate the absolute adaptiveness (*W*_*i*_) for each codon *i*:

$$ {W}_i={\sum}_{j=1}^{n_i}\left(1-{s}_{ij}\right)\ \mathrm{tGC}{\mathrm{N}}_{ij} $$

where *n*_*i*_ is the number of tRNA isoacceptors that recognize the *i*th codon, tGCN_*ij*_ is the gene copy number of the *j*th tRNA that recognizes the *i*th codon, and *s*_*ij*_ is a selective constraint on the efficiency of the codon-anticodon coupling [69]. tRNA gene copy numbers for a mouse (mm10) were retrieved from the GtRNAdb (http://gtrnadb.ucsc.edu, January 2018) [63]. From the *W*_*i*_ values, the relative adaptiveness value *w*_*i*_ of a codon is obtained as:

$$ {w}_i={W}_i/{W}_{\mathrm{max}}\ \mathrm{if}\ {W}_i\ne 0\ \mathrm{or}\ {w}_i={w}_{\mathrm{mean}}\ \mathrm{otherwise} $$

where *W*_max_ is the maximum *W*_*i*_ value, and *w*_mean_ is the geometric mean of all *w*_*i*_. The tRNA adaptation index tAI_g_ of a gene *g* is defined as the geometric mean of the relative adaptiveness values of its codons:

$$ \mathrm{tA}{\mathrm{I}}_{\mathrm{g}}={\left({\prod}_{k=1}^{l_g}{w}_{i_{kg}}\right)}^{1/{l}_{\mathrm{g}}} $$

where *i*_*kg*_ is the codon defined by the *k*th triplet in gene *g* and *l*_g_ is the length of the gene in codons (except the stop codon).

### Model of codon elongation speed

We modeled the codon elongation cycle with the following a simple system of chemical reactions:

$$ {\displaystyle \begin{array}{c}U\overset{k_r}{\to }C\\ {}C+{C}_0\overset{k_{on}}{\to }{C}_1\\ {}{C}_1\overset{k_t}{\to }{C}_2\\ {}{C}_2\overset{k_e}{\to }U+{C}_3\\ {}{C}_3\overset{k_{ep}}{\to }{C}_0\end{array}} $$

where *U* and *C* are the number of uncharged and charged tRNAs, respectively; *C*_*0*_ represents the state where the codon at the A-site of the ribosome is not bound yet by the cognate tRNA; *C*_*1*_ represents the state where the codon is bound to the cognate tRNA at the A-site of the ribosome; *C*_*2*_ represents the state where the codon-cognate tRNA translocated from the A-site to the P-site of the ribosome; and C_3_ represents the state when the specific codon is not yet in the A-site of the ribosome. The reaction rates are as follows: *k*_*r*_, rate of tRNA recharging; *k*_*on*_, rate of codon-cognate tRNA recognition; *k*_*t*_, rate of translocation from the A-site to the P-site; *k*_*e*_, exit rate from P-site, yielding a free codon and an uncharged tRNA; and *k*_*ep*_, rate with which a given codon enters the A-site of the ribosome.

The time that a given codon needs for recognition by the cognate tRNA and translocation is:

$$ {\tau}_{el}={\tau}_{\mathrm{recogn}}+{\tau}_{\mathrm{trans}}=\frac{1}{k_{on}C}+\frac{1}{k_t} $$

Consequently, the elongation speed can be calculated as:

$$ v=\frac{1}{\tau_{el}}=\frac{k_{on}{k}_tC}{k_t+{k}_{on}C} $$

Using the steady-state solution from the system of reactions, we can substitute in *v* the number of charged tRNAs (*C*) with:

$$ C=\frac{1}{2}\left( tRNA-\frac{k_{ep}{k}_r{k}_t+{k}_{ep}{k}_r{k}_e+{k}_{ep}{k}_e{k}_t}{k_{ep}{k}_t+{k}_e{k}_{ep}+{k}_e{k}_t}\;\frac{1}{k_r} codon-\frac{k_e{k}_{ep}{k}_t}{k_{ep}{k}_t+{k}_e{k}_{ep}+{k}_e{k}_t}\;\frac{1}{k_{on}}+\sqrt{{\left( tRNA-\frac{k_{ep}{k}_r{k}_t+{k}_{ep}{k}_r{k}_e+{k}_{ep}{k}_e{k}_t}{k_{ep}{k}_t+{k}_e{k}_{ep}+{k}_e{k}_t}\;\frac{1}{k_r} codon-\frac{k_e{k}_{ep}{k}_t}{k_{ep}{k}_t+{k}_e{k}_{ep}+{k}_e{k}_t}\;\frac{1}{k_{on}}\right)}^2+4\frac{k_e{k}_{ep}{k}_t}{k_{ep}{k}_t+{k}_e{k}_{ep}+{k}_e{k}_t}\;\frac{1}{k_{on}} tRNA}\right) $$

and estimate the expected elongation speed for different values of total tRNAs (= *C*+*U*+*C*_1_+*C*_2_) and codons (=*C*_0_+*C*_1_+*C*_2_+*C*_3_). Assuming the reaction rates are constant for the different codons, the variability in elongation will mostly depend on tRNA availability and codon abundance.

We have performed simulations using the software Wolfram Mathematica v10.0.2.0 (http://www.wolfram.com/mathematica/) and the following reaction rates: *k*_*r*_ = 0.01, *k*_*on*_ = 0.0001, *k*_*t*_ = 1, *k*_*e*_ = 1, and *k*_*ep*_ = 0.001, selected based on [51]. Importantly, the interpretation of th results and conclusions were not affected by modulating these rate constants.

### Gene set enrichment analysis

The tool GSEA v2.2.3 (http://software.broadinstitute.org/gsea/index.jsp) [70] was used to calculate the enrichment of gene sets derived from the KEGG pathway database, Gene Ontology: Biological Processes, and the Hallmark collection. *P* values were estimated by comparing the empirical enrichment score (ES) of a gene set relative to a null distribution of ESs derived from permuting the gene set 1000 times and then adjusted for multiple hypotheses testing [70].

### Statistical analysis

Statistical analysis was performed with Prism 7.0c (GraphPad). For the different statistical tests performed, a *P* < 0.05 was considered significant.

## Supplementary information

**Additional file 1: Figure S1.** mRNAs enriched in different cell-cycle phases display distinct codon bias. **Figure S2.** Codon usage of tissue-specific mRNAs reflects the proliferative capacity of the tissue. **Figure S3.** Growing cells in different serum concentrations does not lead to stress response. **Figure S4.** Quality control of RPF sequencing data. **Figure S5.** Association between codon scores of differentially translated and differentially expressed genes is consistent across growing conditions. **Figure S6.** Characterization of cell-cycle phases and global protein synthesis in cell lines harboring distinct reporters grown in different conditions. **Figure S7.** Analysis of tRNA expression in cells grown in different media.

**Additional file 2: Table S1.** Codon usage of different gene sets (G1-specific, G2M-specific and All genes).

**Additional file 3: Table S2.** Codon scores across different experiments.

**Additional file 4: Table S3.** Differential expression analysis of mRNA and ribosome occupancy (10% vs 1% FCS).

**Additional file 5: Table S4.** Differential expression analysis of mRNA and protein levels (10% vs 1% FCS).

**Additional file 6: Table S5.** tRNA A34I modification analysis.

**Additional file 7: Table S6.** Primer, adapter and coding sequences.

**Additional file 8.** Review history.

## Data Availability

Sequencing data from this study have been submitted to NCBI under the accession number PRJNA472989 [71]. The mass spectrometry proteomics data have been deposited to the ProteomeXchange Consortium via the PRIDE [72] partner repository with the dataset identifier PXD016034 [73]. mRNAs expression data for 15 different mouse tissues were retrieved from [67]. The source code to replicate the analysis presented in this study is available from Zenodo at 10.5281/zenodo.3612157 [74].
