# Supplementary material for: A rare codon-based translational program of cell proliferation
Source: Genome Biol. 2020 Feb 27;21:44. doi: 10.1186/s13059-020-1943-5 (PMC7045563; doi:10.1186/s13059-020-1943-5)
Supplement: Supplementary file 1 — Additional file 1: Figure S1. mRNAs enriched in different cell-cycle phases display distinct codon bias. Figure S2. Codon usage of tissue-specific mRNAs reflects the proliferative capacity of the tissue. Figure S3. Growing cells in different serum concentrations does not lead to stress response. Figure S4. Quality control of RPF sequencing data. Figure S5. Association between codon scores of differentially translated and differentially expressed genes is consistent across growing conditions. Figure S6. Characterization of cell-cycle phases and global protein synthesis in cell lines harboring distinct reporters grown in different conditions. Figure S7. Analysis of tRNA expression in cells grown in different media. [file 13059_2020_1943_MOESM1_ESM.pdf]

Additional file 1: Fig. S1-S7

A rare-codon-based translational program of cell proliferation

Joao C. Guimaraes[1,*], Nitish Mittal[1], Alexandra Gnann[1,2], Dominik Jedlinski[1], Andrea Riba[1,3], Katarzyna Buczak[4], Alexander Schmidt[4] and Mihaela Zavolan[1,*]

[1]Computational and Systems Biology, Biozentrum, University of Basel, Klingelbergstrasse 50-70, 4056 Basel, Switzerland

[2]Department of Biomedicine, University of Basel / University Hospital Basel, Hebelstrasse 20, 4031 Basel, Switzerland

[3]Institut de Génétique et de Biologie Moléculaire et Cellulaire, Université de Strasbourg, 1 rue Laurent Fries 67404, Illkirch CEDEX, France

[4]Proteomics Core Facility, Biozentrum, University of Basel, Klingelbergstrasse 50-70, 4056 Basel, Switzerland

*Correspondence to: J.C.G. (joaoguima@gmail.com) or M.Z. (mihaela.zavolan@unibas.ch).

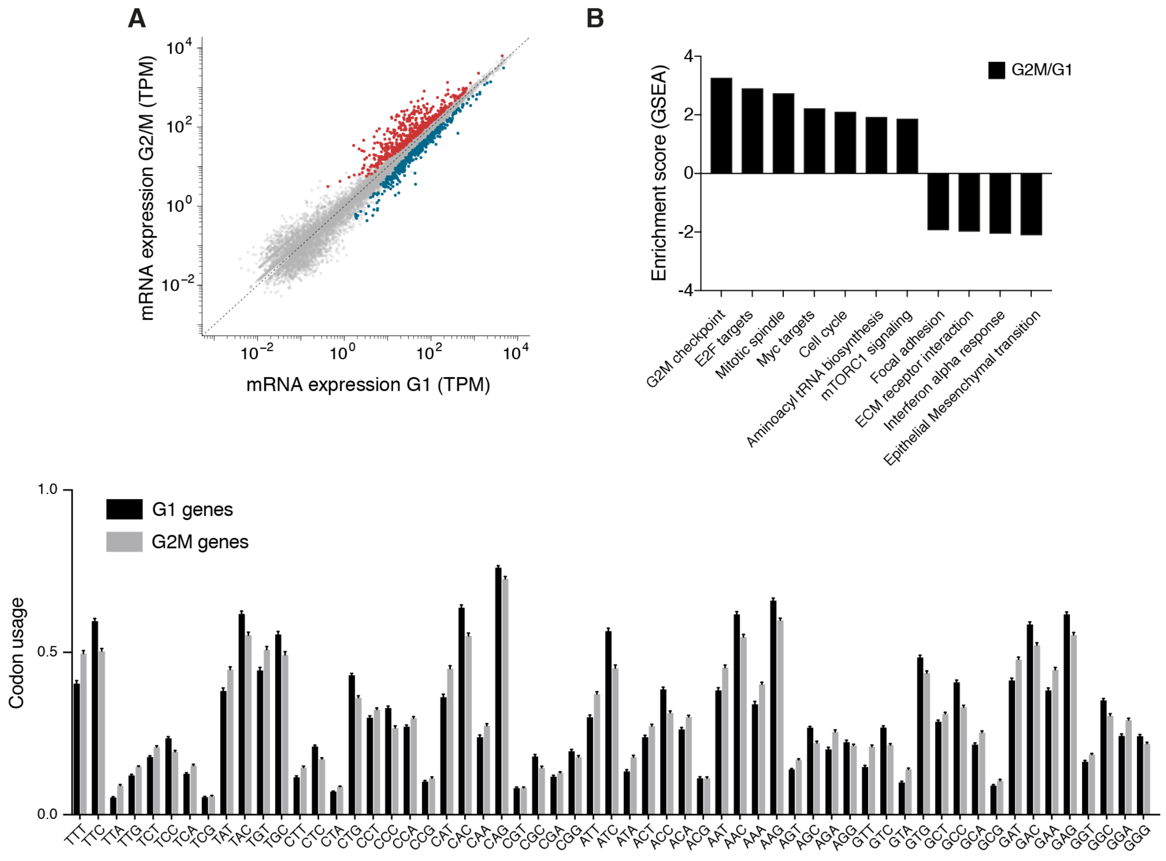

Fig. S1 mRNAs enriched in different cell-cycle phases display distinct codon bias. (**A**) Scatter plot of mRNA expression levels (log10 transcript counts per million, TPM) in G2/M and G1 cell-cycle phases. Shown are mean expression values for each transcript in each cell-cycle phase (n=2). mRNAs significantly upregulated or downregulated (False discovery rate, FDR<0.01) in G2/M phase are shown in red and blue, respectively. The dashed line indicates equal abundances in the two different conditions. (**B**) Normalized enrichment score (ES) for different categories of genes, derived from gene set enrichment analysis (GSEA) of gene expression in the G2/M and G1 cell-cycle phases. For all the pathways depicted, $P<0.05$. *P*-values were calculated by comparing the empirical ES of a gene category to a null distribution of ESs derived from permuting the gene set, and then adjusting for multiple hypotheses testing. (**C**) Mean (+/- s.e.m.) relative of usage of each codon within its synonymous set, in genes specifically expressed in the G1 and G2/M cell-cycle phases.

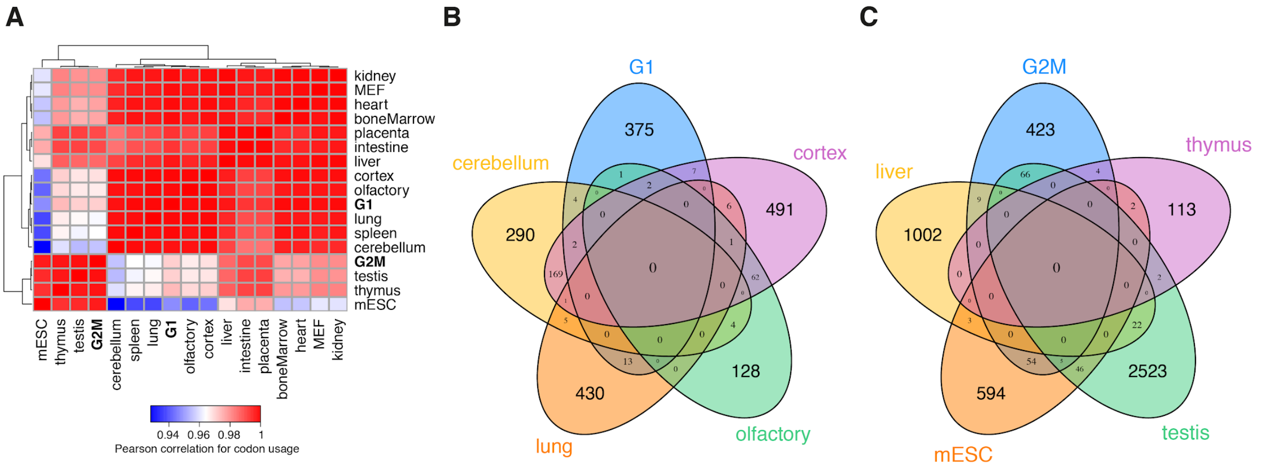

Fig. S2 Codon usage of tissue-specific mRNAs reflects the proliferative capacity of the tissue.

(**A**) Heatmap depicting the matrix of Pearson correlation coefficients comparing the codon usage of mRNAs enriched in the G1 and G2/M cell-cycle phases, and in 15 different mouse tissues. mRNAs enriched in the G1 and G2/M cell-cycle phases were identified as described in the main text, and mRNAs with higher expression in a specific tissue relative to the average over all tissues were determined from Ref. [63]. (**B, C**) Venn diagram of tissue-specific mRNAs displaying a codon usage similar to G1 (**B**) or G2/M genes (**C**).

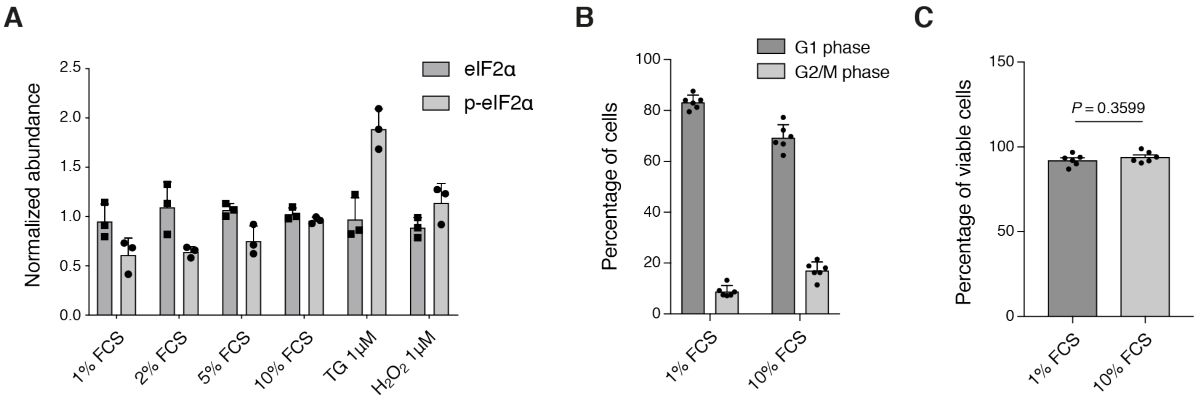

Fig. S3 Growing cells in different serum concentrations does not lead to stress response. (**A**) Mean (+/- s.e.m.) abundance of eIF2α and phosphorylated eIF2α (p-eIF2α) estimated from western blots after normalization using the loading control tubulin, for cells growing in either 1%, 2%, 5% or 10% FCS, as well as in two control conditions triggering stress responses (Thapsigargin (TG) and hydrogen peroxide) (n=3). (**B**) Mean (+/- s.e.m.) percentage of NIH-3T3 cells in the G1 and G2/M phases of the cell cycle, as determined by Hoechst staining for DNA content, for cells grown in either 1% or 10% FCS (n=6). (**C**) Mean (+/- s.e.m.) percentage of viable cells for cells grown in either 1% or 10% FCS (n=6). Shown are also the *P*-values determined by the non-parametric Mann-Whitney U test.

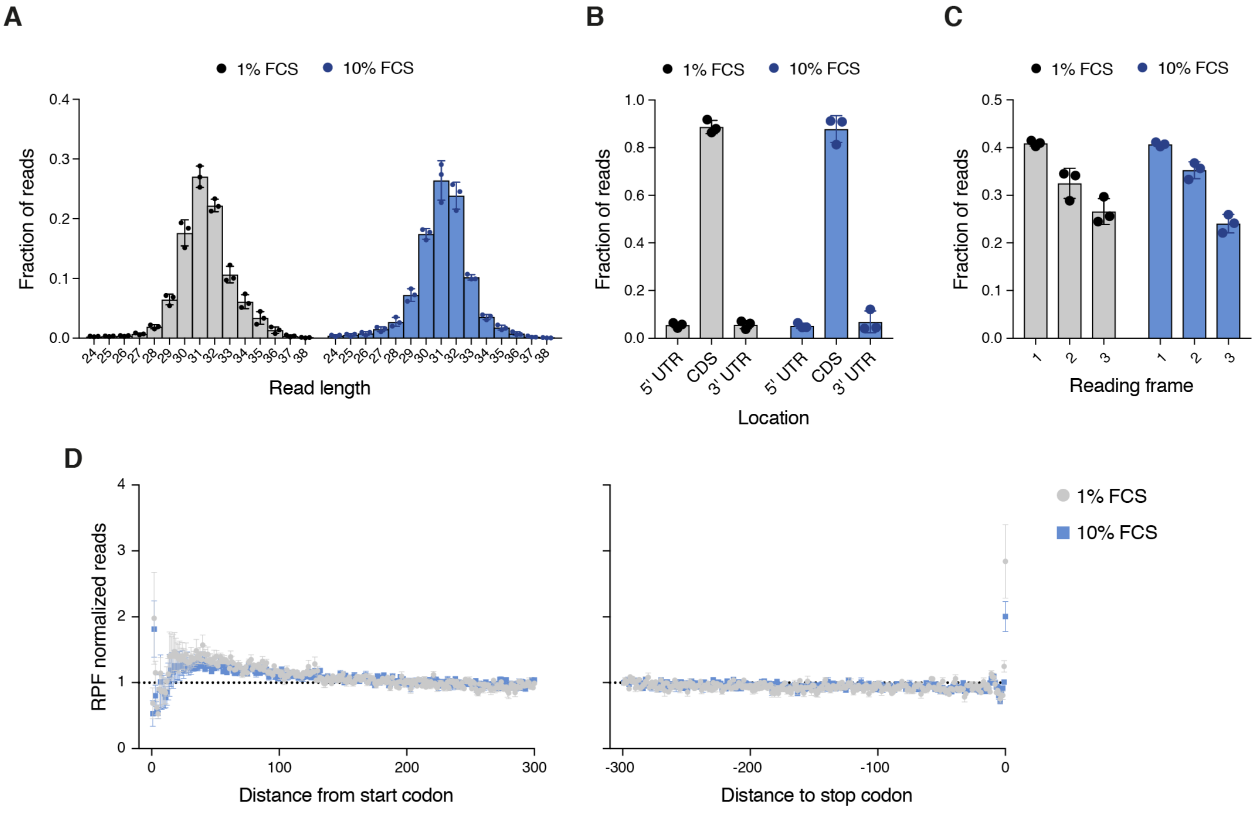

Fig. S4 Quality control of RPF sequencing data.

(**A**) Mean (+/- s.e.m.) fraction of ribosome protected fragments (RPF) reads of the indicated lengths in samples from cells grown in either 1% (gray) or 10% (blue) FCS (n=3). (**B**) Mean (+/- s.e.m.) of the proportion of RPF reads whose inferred A-site mapped to either 5' untranslated region (UTR), coding sequence (CDS) or 3'UTR in samples from cells grown in either 1% (gray) or 10% (blue) FCS (n=3). (**C**) Mean (+/- s.e.m.) fraction of RPF reads whose inferred A-site mapped to each of the three reading frames (1 represents the first codon position) in samples from cells grown in either 1% (gray) or 10% (blue) FCS (n=3). (**D**) Metagene analysis of mean (+/- s.e.m.) RPF reads at the 5' and 3' end of coding sequences in samples from cells grown in either 1% (gray) or 10% (blue) FCS (n=3). RPF reads per codon (based on the inferred A-site) in a given CDS were individually normalized by the mean number of reads within the respective CDS, and then averaged across all genes.

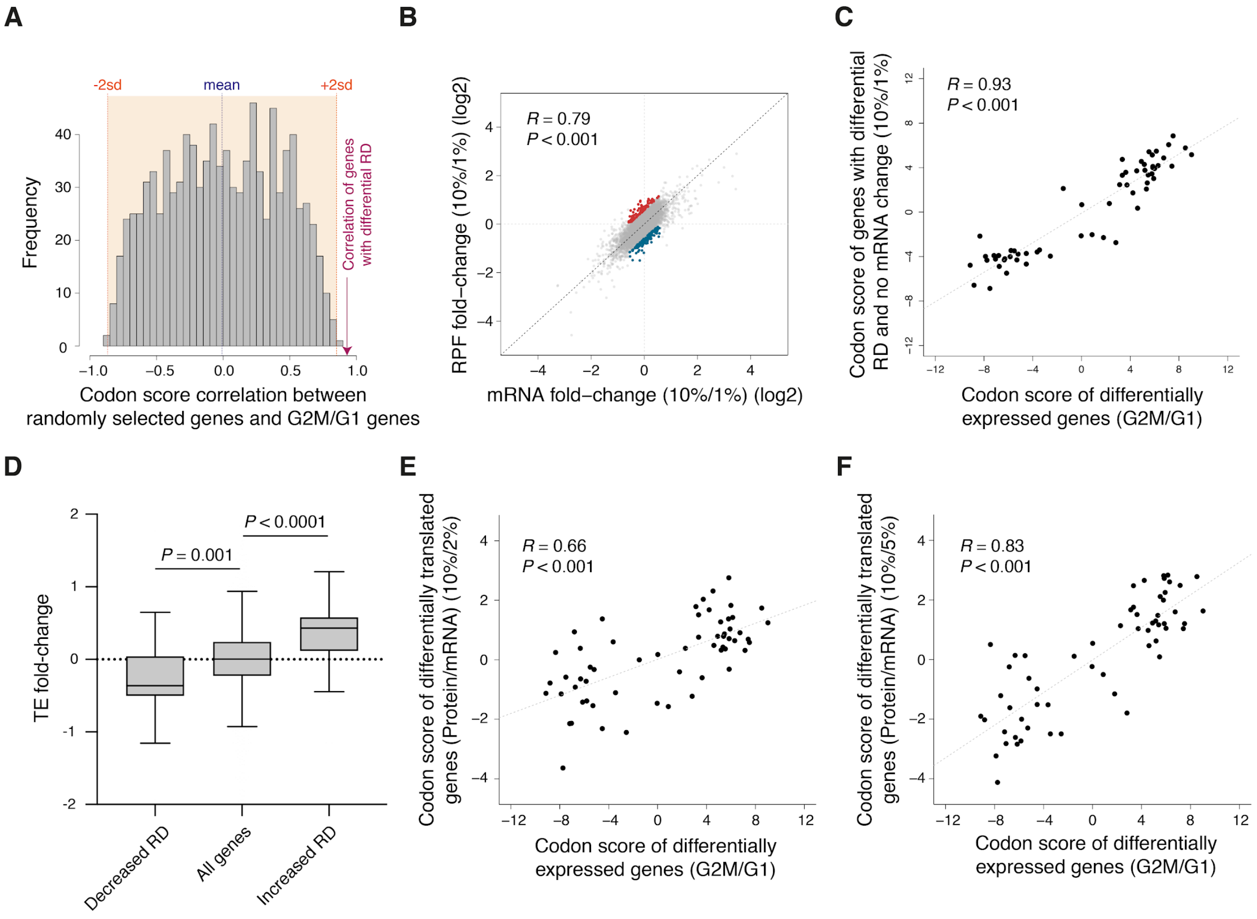

Fig. S5 Association between codon scores of differentially translated and differentially expressed genes is consistent across growing conditions.

(**A**) Distribution of Pearson correlation coefficients between codon scores of randomly selected genes and differentially expressed genes in G2M/G1 (n=1,000). Blue and orange vertical lines indicate the mean and +/-2 standard deviations, respectively. The purple arrow shows the correlation found between the codon scores of genes with differential ribosome density (RD) and differential expressed genes in G2M/G1. (**B**) Scatter plot of the log2 fold-changes in mRNA and ribosome protected fragments (RPF) in cells grown in 10% relative to 1% FCS. Shown are mean values computed for each transcript from mRNA (n=4) and RPF (n=3) replicates. Transcripts upregulated and downregulated (changed more than 50% in either direction) at ribosome density level (RD, defined as $RPF_{fold\text{-}change}$ / $mRNA_{fold\text{-}change}$) but unchanged (change less than 50%) at mRNA level, are shown in red and blue, respectively. Shown is the Pearson correlation coefficient and respective *P*-value. The dashed line indicates equal change in mRNA and RPF levels. (**C**) Scatter plot of per-codon scores among genes that are differentially expressed

between G2/M and G1 cell-cycle phases and genes with differential ribosome density (RD, defined as $RPF_{fold\text{-}change}$ / $mRNA_{fold\text{-}change}$) but no change in mRNA level when cells are grown in 10% relative to 1% FCS. Shown is the Pearson correlation coefficient and respective *P*-value. The dashed line indicates the linear regression between the two estimates. (**D**) Boxplots showing the distribution of log2 fold-changes in translation efficiency (defined as the residuals of the linear regression between $Protein_{fold\text{-}change}$ and $mRNA_{fold\text{-}change}$, see **Fig. 2H**) between cells grown in 10% relative to 1% FCS, for genes with decreased or increased ribosome density (RD, see **Fig. 2D**) between these two conditions. Shown are the *P*-values determined by the Dunn's multiple comparisons test post hoc the non-parametric Kruskal-Wallis test ($P<0.0001$). Boxes extend from the 25th to 75th percentiles (inter-quartile range (IQR)), horizontal lines represent the median, whiskers indicate the lowest and highest datum within 1.5*IQR from the lower and upper quartiles, respectively. (**E**, **F**) Scatter plot of per-codon scores among genes that are differentially expressed between G2/M and G1 cell-cycle phases and genes that are differentially translated, as inferred from comparing protein and mRNA fold-changes, when cells are grown in 10% relative to 2% (**E**) or 5% (**F**) FCS. Shown are also the Pearson correlation coefficient and respective *P*-values. The dashed lines indicate the linear regression between the two estimates.

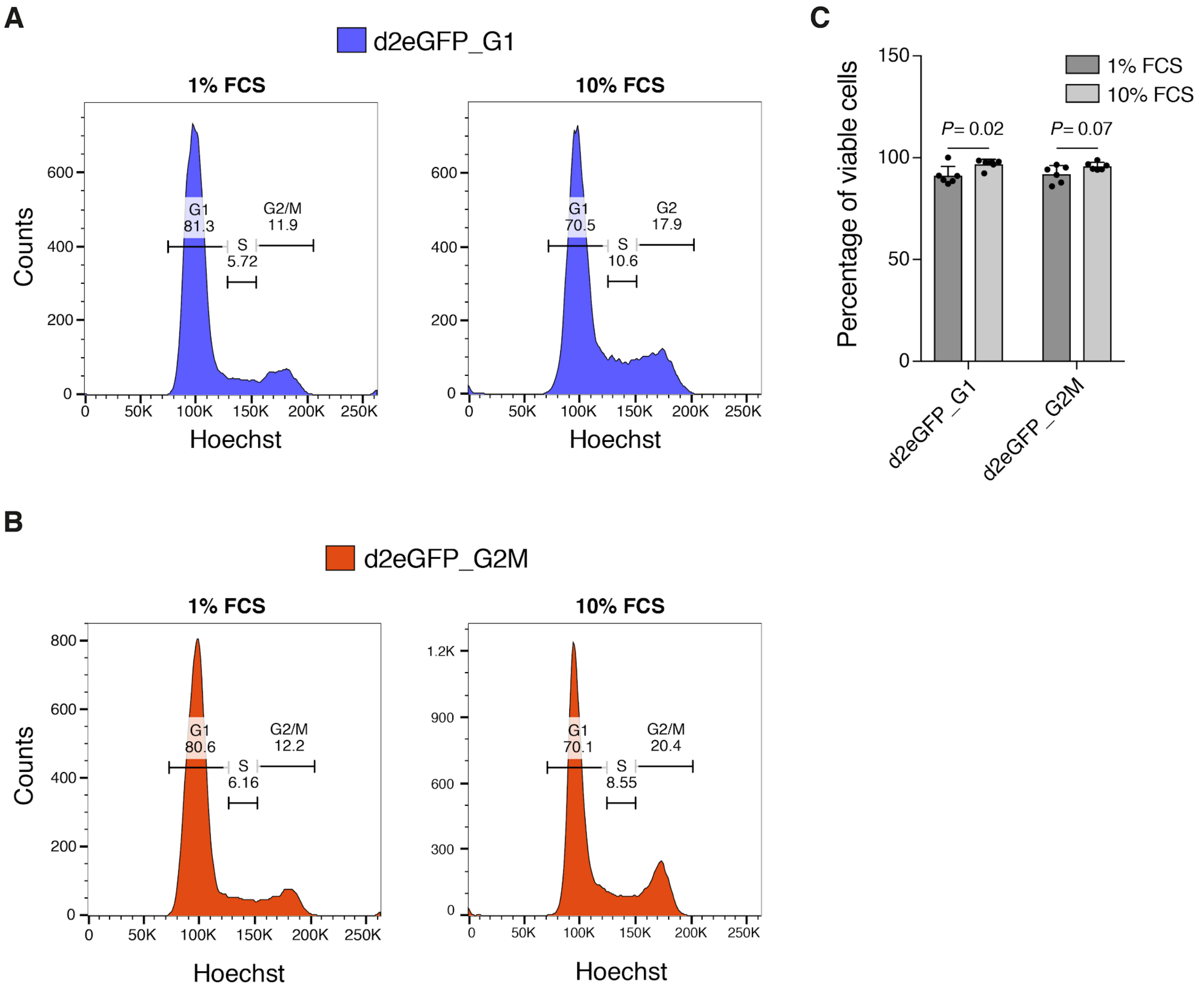

Fig. S6 Characterization of cell-cycle phases and global protein synthesis in cell lines harboring distinct reporters grown in different conditions.

(**A-B**) Representative result of the flow cytometry analysis of Hoechst intensity (DNA content) in the two cell lines, d2eGFP_G1 (**A**) and d2eGFP_G2M (**B**), grown in either 1% or 10% FCS. The percentage of cells in each cell-cycle phase is depicted. (**C**) Mean (+/- s.e.m.) percentage of viable cells for the two cell lines, d2eGFP_G1 and d2eGFP_G2M, grown in either 1% or 10% FCS (n=6). Shown are also the *P*-values determined by the non-parametric Mann-Whitney U test.

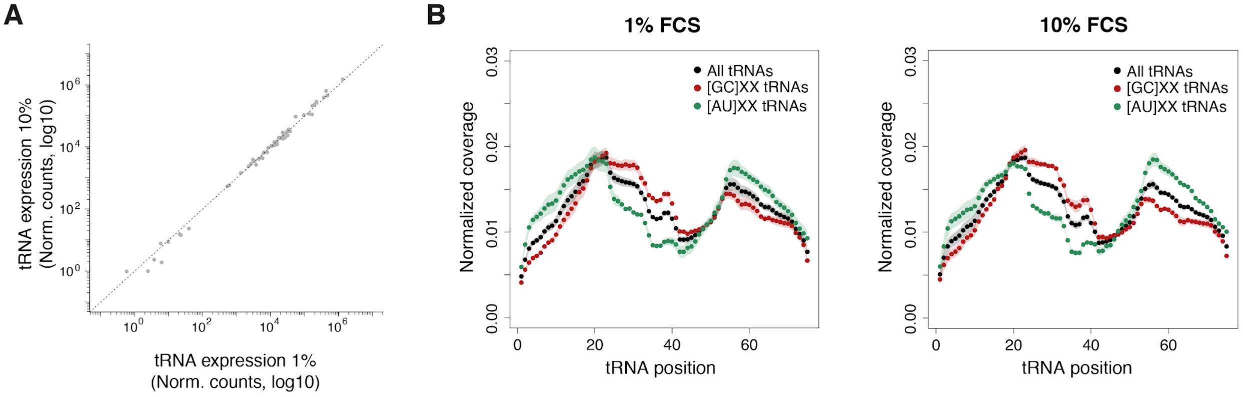

Fig. S7 Analysis of tRNA expression in cells grown in different media.

(**A**) Scatter plot of tRNA gene (aggregated by isoacceptors) expression levels (log10, library size-normalized counts) in cells grown in either 10% or 1% FCS. Shown are mean expression values for each tRNA isoacceptor gene (n=3). There were no isoacceptor genes significantly upregulated or downregulated (False discovery rate, FDR < 0.01). The dashed line indicates equal abundances in the two conditions. (**B**) Mean (+/- s.d.) fraction of reads covering all tRNA genes, or tRNA genes containing anticodons with G/C or A/U at the first position, in cells grown in either 1% (left) or 10% (right) FCS.
