# Supplementary material for: A rare codon-based translational program of cell proliferation
Source: Genome Biol. 2020 Feb 27;21:44. doi: 10.1186/s13059-020-1943-5 (PMC7045563; doi:10.1186/s13059-020-1943-5)
Supplement: Supplementary file 8 — Additional file 8. Review history. [file 13059_2020_1943_MOESM8_ESM.docx]

Review History

**First round of review**

**Reviewer 1**

**Are you able to assess all statistics in the manuscript, including the appropriateness of statistical tests used?**

Yes, and I have assessed the statistics in my report.

**Comments to author:**

The authors are investigating a very interesting topic, how translation and tRNAs control gene regulation and cells stage. There is a lot of controversy about the role of tRNAs affecting gene expression between proliferating and nondifferentiated cells and there are growing evidences that tRNA/translation can affect gene expression in a codon dependent manner. A so, synonymous codons are not truly "silence". While the topic is extremely interesting, the field need to be extremely rigorous and conclusions should be arising from clear experimental results.

The authors have used a very nice system to observe that proliferating cells displayed mRNA enriched in rare codon. And while this is a very interesting observation there are some comments that I would like to mention.

In Figure 2, the authors identify hundreds of mRNAs which translation efficiency change more that 50%, however, those genes also display a huge change in mRNA level, maybe stability. While translation efficiency "normalizes" the mRNA level there is a bias between mRNA level and TE, therefore is it not fair to say that those genes are more efficiency translated when the differences at mRNA are so strong. The authors can take a random group of genes that contain the same amount of RNA and then ask the TE of their mRNA compare to that control group.

The authors, very nicely wrote in the introduction that synonymous codon can affects mRNA stability, protein folding and protein function. However, the reporters that they used (Fig 3) can easily be affected by protein folding, nucleotide composition, etc. I think, it is crucial to develop a large variety of reporters controlling for a lot of variables and/or using alternative readout or alternative reporters to validate their results.

Then for the proposed model (Fig 4), one of the components is the number of charged tRNAs, however there is not experimental evidence that the charged tRNA might change nor play any role here. It is interesting to speculate the charged-tRNA or ready to go tRNA is important, but experimental evidences are required. I completely agree that measurement of tRNA is hard, not very reproducible and also rise a lot of controversy. Also, the number of ribosomes can affects the model.

In sum, while the author found very interesting correlations but those correlations and observation does not fully support very strong conclusion such as "Our findings provide direct evidence that codon bias is exploited to yield dynamic changes in translation efficiency, and that rare codons, rather than persisting passively in genomes due to reduced selection for translation efficiency, may be actively used to modulate translation rates." For this strong conclusion, I honestly think that tRNA level need to be perturbed (technically very hard) and evidence very clear arrest or lost proliferation ability.

**Reviewer 2**

**Are you able to assess all statistics in the manuscript, including the appropriateness of statistical tests used?**

No, I do not feel qualified to assess the statistics

**Comments to author:**

The manuscript entitled, "A rare-codon-based translational program of cell proliferation" presents an interesting and novel argument for the differential effects of rare codons on gene expression in differing states of cellular proliferation. The authors begin with RNA sequencing experiments to identify genes differentially expressed in cells in G1 or G2/M phases of the cell-cycle. These findings suggest a different expression profiles and differences in codon usage bias in the highly expressed transcripts in each phase. In figure 1 of the manuscript, the authors suggest cells in G2/M phase preferentially use codons which have an A or a U as the third base. The authors suggest a possible decrease in structure formation in highly expressed G2/M transcripts as measured by free energy of folding suggesting an ulterior mechanism of post-transcriptional gene regulation. The authors performed tRNA sequencing on cells utilizing the Hydro-seq methodology to determine levels of tRNA abundance. From this data the authors concluded G2/M phase transcripts were not well adapted to the tested tRNA pools. The authors then correlated codon usage of different mouse tissues from existing RNA sequencing data to proliferative capacity and concluded a possible association exists between the usage of codons enriched in G2/M phase from previous studies and tissue-specific mRNA expression. The authors then begin to study the effects of proliferative and non-proliferative cell states on fluorescent reporters enriched in G1 codons or G2/M specific rare codons. The authors utilized NIH3T3 cells cultured in 10% and 1% serum to model proliferative and non-proliferative populations respectively. Utilizing these reporter assays, the authors determined the increase in expression of the G2/M reporter in cells cultured 10% serum was secondary to an increased ability of cells to decode rare codons. The authors conclude the manuscript with a model suggesting differential changes in translational efficiency over common and rare codons as levels of their cognate tRNAs approach saturation. In common codons the tRNA levels are at or approaching saturation, while the tRNA levels of rare codons are limiting. The limiting tRNA levels for rare codons allows for a greater potential increase in translation efficiency of rare codons compared to more common codons in a proliferative cellular state.

If this proposed model proves to be accurate, this would be a novel finding in the field of translational control, However, it is my recommendation this manuscript is not considered for publication in Genome Biology at this time due to the lack of critical controls underlying the methodologies of this paper. Outlined below are a number of experiments essential to supporting the authors' interpretation of the data provided. I would be happy to complete a more thorough review if these experiments were performed and data provided.

The authors utilized a reduced serum model for altering proliferative capacity NIH3T3 cells. This possibly introduces many confounding variables which were not tested or controlled for adequately by the authors of this manuscript. It is important for the authors to assess stress responses following culture in reduced (1%) serum. While viability and global incorporation of labeled methionine are helpful, the authors did not perform the appropriate controls for their methionine incorporation experiments in comparing cells cultured in 1% serum to cells cultured in 10% serum. The authors did not provide data for cells cultured in 1% serum. In addition to these experiments, it would be critical to perform western blots for phosphorylation of eIF2a. Phosphorylated eIF2 has been shown to alter expression of certain transcripts (PMID: 24648495) and reduce global protein synthesis. This could mask any differences between the GFP_G1 and GFP_G2M transcripts if global protein synthesis was decreased. If results from methionine incorporation experiments and blotting for p-eIF2a conclude there is no difference between cells cultured in 10% or 1% serum, this would significantly reinforce the authors' claim of an increase in translation of the GFP_G2M transcripts relative to the GFP_G1 transcript.

Based upon the description provided by the authors in the materials and methods section, the methodology utilized to determine tRNA abundance does not include an internal control capable of capturing global changes in tRNA abundance. In the event cells cultured in 1% FCS decrease general transcription of RNA polymerase III transcripts, global decreases in tRNA transcription and subsequent maturation cannot not be determined. This may confound the results determined in figure 3 and 4 which the authors interpreted as a shift in translation efficiency over rare codons. This interpretation may be misguided. The authors need to provide evidence suggesting the change in translation of the d2eGFP_G2M construct in cells cultured in 1% and 10% is not secondary to a global shift in tRNA abundance and/or a stress response. To control for global changes in tRNA abundance, the introduction of an RNA spike-in to the tRNA sequencing protocol would help to normalize tRNA abundance.

In addition to lack of an internal control, it was unclear based upon the information provided in the materials and methods section if the authors sorted cells from G1 and G2/M phases to determine if distinct pools of tRNA exist during cell-cycle phases. The majority of any heterogeneous cell population will be in G1 phase, therefore, the tRNA pools will reflect this dominant cell population. It would be critical for the authors to determine the presence or absence of a functional tRNA pool in G2/M phase which is distinct from G1 phase. The authors need to repeat the tRNA sequencing experiments with sorted G1 and G2/M cells if they are to claim a difference in tAI between G1 and G2/M transcripts.

**Authors Response**

**Point-by-point responses to the reviewers’ comments:**

Reviewer #1:

The manuscript entitled, "A rare-codon-based translational program of cell proliferation" presents an interesting and novel argument for the differential effects of rare codons on gene expression in differing states of cellular proliferation. The authors begin with RNA sequencing experiments to identify genes differentially expressed in cells in G1 or G2/M phases of the cell-cycle. These findings suggest a different expression profiles and differences in codon usage bias in the highly expressed transcripts in each phase. In figure 1 of the manuscript, the authors suggest cells in G2/M phase preferentially use codons which have an A or a U as the third base. The authors suggest a possible decrease in structure formation in highly expressed G2/M transcripts as measured by free energy of folding suggesting an ulterior mechanism of post-transcriptional gene regulation. The authors performed tRNA sequencing on cells utilizing the Hydro-seq methodology to determine levels of tRNA abundance. From this data the authors concluded G2/M phase transcripts were not well adapted to the tested tRNA pools. The authors then correlated codon usage of different mouse tissues from existing RNA sequencing data to proliferative capacity and concluded a possible association exists between the usage of codons enriched in G2/M phase from previous studies and tissue-specific mRNA expression. The authors then begin to study the effects of proliferative and non-proliferative cell states on fluorescent reporters enriched in G1 codons or G2/M specific rare codons. The authors utilized NIH3T3 cells cultured in 10% and 1% serum to model proliferative and non-proliferative populations respectively.

Utilizing these reporter assays, the authors determined the increase in expression of the G2/M reporter in cells cultured 10% serum was secondary to an increased ability of cells to decode rare codons. The authors conclude the manuscript with a model suggesting differential changes in translational efficiency over common and rare codons as levels of their cognate tRNAs approach saturation. In common codons the tRNA levels are at or approaching saturation, while the tRNA levels of rare codons are limiting. The limiting tRNA levels for rare codons allows for a greater potential increase in translation efficiency of rare codons compared to more common codons in a proliferative cellular state.

If this proposed model proves to be accurate, this would be a novel finding in the field of translational control, However, it is my recommendation this manuscript is not considered for publication in Genome Biology at this time due to the lack of critical controls underlying the methodologies of this paper. Outlined below are a number of experiments essential to supporting the authors' interpretation of the data provided. I would be happy to complete a more thorough review if these experiments were performed and data provided.

The authors utilized a reduced serum model for altering proliferative capacity NIH3T3 cells. This possibly introduces many confounding variables which were not tested or controlled for adequately by the authors of this manuscript. It is important for the authors to assess stress responses following culture in reduced (1%) serum. While viability and global incorporation of labeled methionine are helpful, the authors did not perform the appropriate controls for their methionine incorporation experiments in comparing cells cultured in 1% serum to cells cultured in 10% serum. The authors did not provide data for cells cultured in 1% serum. In addition to these experiments, it would be critical to perform western blots for phosphorylation of eIF2a. Phosphorylated eIF2 has been shown to alter expression of certain transcripts (PMID: 24648495) and reduce global protein synthesis. This could mask any differences between the GFP_G1 and GFP_G2M transcripts if global protein synthesis was decreased. If results from methionine incorporation experiments and blotting for p-eIF2a conclude there is no difference between cells cultured in 10% or 1% serum, this would significantly reinforce the authors' claim of an increase in translation of the GFP_G2M transcripts relative to the GFP_G1 transcript.

*We thank the reviewer for the constructive suggestions. We have performed additional experiments to address the issues raised. First, we carried out western blot analysis to assess the level of phosphorylation of eIF2a, and found no increase in this stress marker in cells cultured at lower serum levels (Figure 2B, C). Second, we have measured the global incorporation of methionine analog in both 1% and 10% FCS as suggested by this reviewer (Figures 3G and 4B). This revealed a similar behavior of the reporter constructs as well as wild-type NIH3T3 cells.*

Based upon the description provided by the authors in the materials and methods section, the methodology utilized to determine tRNA abundance does not include an internal control capable of capturing global changes in tRNA abundance. In the event cells cultured in 1% FCS decrease general transcription of RNA polymerase III transcripts, global decreases in tRNA transcription and subsequent maturation cannot not be determined. This may confound the results determined in figure 3 and 4 which the authors interpreted as a shift in translation efficiency over rare codons. This interpretation may be misguided. The authors need to provide evidence suggesting the change in translation of the d2eGFP_G2M construct in cells cultured in 1% and 10% is not secondary to a global shift in tRNA abundance and/or a stress response. To control for global changes in tRNA abundance, the introduction of an RNA spike-in to the tRNA sequencing protocol would help to normalize tRNA abundance.

*As mentioned above, we have assessed the level of phosphorylated eIF2a, and found no increase when cells were cultured in 5%, 2% or 1% FCS (Figure 2B, C). Thus, there is no indication that changes in translation of mRNAs with rare codons represent a secondary effect of cellular stress. Furthermore, we have also quantified genome-wide translation efficiencies combining proteomics and transcriptomics measurements, not only in cells growing in 1% compared to 10% serum, but also in cells grown in more ‘permissive’ culture conditions, with 2% or 5% serum. These experiments further confirmed the increased translation efficiency of mRNAs enriched in rare codons in 10% compared to lower serum concentrations (Figures 2H, I, J and Figures S5D, E). We believe these data convincingly show that the observed association between codon bias and translation efficiency is not due to a stress response.*

*We also agree with the reviewer that the absence of RNA spike-ins in our tRNA sequencing experiment limits our ability to infer global shift in tRNA abundance. However, our gene set enrichment analysis (Figure 4A) does indicate a global increase in translation capacity in the 10% serum condition, and work in yeast showed that the tRNA abundance increases ~2-fold in the G2 compared to other phases of the cell cycle [Frenkel-Morgenstern et al. MSB 2012]. Given these observations and for time and resource constraints we did not repeat the sequencing experiment using RNA spike-ins. In revising our manuscript, we also obtained experimental evidence that proliferating cells show slightly increased levels of tRNA aminoacylation levels, which we determine by quantifying charged/uncharged abundances of a specific tRNA (tRNA-Lys-UUU) (Figure 4F, G) in northern blots.*

*Regarding the reviewer’s question of whether global changes in tRNA abundance may be at the origin of the translational changes, we indeed believe this to be the case. Why a global change in tRNA levels would preferentially boost the translation of transcripts enriched in rare codons (which we show both by ribosome profiling and proteomics measurements in cells with various proliferation rates) is a central question that we tried to answer, and we believe that our data (Figure 4 and Figure 5) suggest a model where mRNAs that are elongation-limited in cells with low proliferation rate (i.e. those enriched in rare codons) have a stronger boost in elongation upon a global increase in tRNA abundance compared to those transcripts that are not elongation limited.*

In addition to lack of an internal control, it was unclear based upon the information provided in the materials and methods section if the authors sorted cells from G1 and G2/M phases to determine if distinct pools of tRNA exist during cell-cycle phases. The majority of any heterogeneous cell population will be in G1 phase, therefore, the tRNA pools will reflect this dominant cell population. It would be critical for the authors to determine the presence or absence of a functional tRNA pool in G2/M phase which is distinct from G1 phase. The authors need to repeat the tRNA sequencing experiments with sorted G1 and G2/M cells if they are to claim a difference in tAI between G1 and G2/M transcripts.

*We agree with this reviewer that ideally one would quantify tRNA abundances in cells sorted from different cell-cycle phases, however it is extremely challenging to get enough material for under-represented populations (in this case G2/M) to perform sequencing experiments.*

*For this reason, we decided to perform the other experiments culturing cells in different serum levels, where we can modulate the rate of cell division (i.e. the proportion of cells G2/M) while getting sufficiently large cell numbers to perform high-throughput experiments (transcriptomics, ribosome occupancy, proteomics and tRNA sequencing). While we agree with the reviewer that in all of these experiments the G1 phase dominates in the cell population, there is a significant difference in the proportion of cells in G2/M between culture conditions (Figure 2A), and this difference was sufficient for us to detect changes in the translation efficiency of specific mRNAs (Figures 2D, H). We thus believe that if significant deviations in the abundance of individual tRNAs underlied the observed differences in translation, we would have been able to detect them. Finally, the difference in tAI between G1 and G2/M transcript that we reported (Figures 1F, H) was predicted computationally by estimating tRNA abundances using corresponding genome copy numbers, a widely used approach to determine the relative adaptiveness of mRNAs to cellular pools. Ideally, these estimates would be inferred from empirical data, but as we mentioned, obtaining such data is extremely challenging.*

Reviewer #2:

The authors are investigating a very interesting topic, how translation and tRNAs control gene regulation and cells stage. There is a lot of controversy about the role of tRNAs affecting gene expression between proliferating and nondifferentiated cells and there are growing evidences that tRNA/translation can affect gene expression in a codon dependent manner. A so, synonymous codons are not truly "silence". While the topic is extremely interesting, the field need to be extremely rigorous and conclusions should be arising from clear experimental results.

The authors have used a very nice system to observe that proliferating cells displayed mRNA enriched in rare codon. And while this is a very interesting observation there are some comments that I would like to mention.

In Figure 2, the authors identify hundreds of mRNAs which translation efficiency change more that 50%, however, those genes also display a huge change in mRNA level, maybe stability. While translation efficiency "normalizes" the mRNA level there is a bias between mRNA level and TE, therefore is it not fair to say that those genes are more efficiency translated when the differences at mRNA are so strong. The authors can take a random group of genes that contain the same amount of RNA and then ask the TE of their mRNA compare to that control group.

*We thank the reviewer for this suggestion, which we followed, performing a similar analysis to the one shown in Figure 2D but only on genes whose mRNA expression does not change significantly (mRNA changed less than 50% in either direction). This analysis confirmed that there is an association between improved translation efficiency and rare codons even when we only consider mRNAs whose levels did not change significantly between resting and proliferating cells (Figure S5A, B).*

The authors, very nicely wrote in the introduction that synonymous codon can affects mRNA stability, protein folding and protein function. However, the reporters that they used (Fig 3) can easily be affected by protein folding, nucleotide composition, etc. I think, it is crucial to develop a large variety of reporters controlling for a lot of variables and/or using alternative readout or alternative reporters to validate their results.

*While we agree with the reviewer that testing a larger collection of reporters would be one approach to validate our results, we believe that our data on genome-wide measurement of ribosome densities provide an independent dataset validating our hypothesis. For this revision, we have additionally performed proteomics measurements in cell growing in different conditions (1%, 2%, 5% and 10% FCS), which further validate that transcripts whose translation changes (i.e. protein fold-change normalized by mRNA fold-change changed more than 50%) between conditions share the codon bias that we have initially identified in our analysis of codon usage across the cell cycle.*

Then for the proposed model (Fig 4), one of the components is the number of charged tRNAs, however there is not experimental evidence that the charged tRNA might change nor play any role here. It is interesting to speculate the charged-tRNA or ready to go tRNA is important, but experimental evidences are required. I completely agree that measurement of tRNA is hard, not very reproducible and also rise a lot of controversy. Also, the number of ribosomes can affects the model.

*We thank the reviewer for this comment, we had provided evidence that genes involved in tRNA biogenesis and metabolism were strongly upregulated in rapidly proliferating cells (Figure 4A) and for this revision we decided to follow the suggestion to measure tRNAs using northern blot. As pointed out by the reviewer, these are extremely challenging and time-consuming experiments and for this reason we were only able to measure reliably one tRNA (tRNA-Lys-UUU), for which we present quantifications in Figure 4F, G. We were able to find a slight increase in the aminoacylation levels of this tRNA in cells grown in 10% compared to 1% FCS. We acknowledge that our new experiments do not extensively answer the reviewer’s question, but we note that we have been trying to establish these experiments for months and data on charged/uncharged tRNAs has been extremely difficult to collect.*

*Furthermore, we would like to point out that a previous study in yeast [Frenkel-Morgenstern, et al., MSB, 2012] did find that the total tRNA abundance is ~2-fold higher in the G2 phase compared to other phases of the cell cycle. We believe that other independent analyses presented in our paper, such as the upregulation of tRNA biogenesis and metabolism inferred from mRNA-seq data do provide support for the model that we propose. We also emphasize that our manuscript provides very extensive and rather surprising data on the genome-wide translational efficiency changes in cells with different proliferation rates. While we propose one model that may explain these data, we believe that the data itself would be of great interest in the field and would prompt evaluation of additional models.*

*Regarding the number of ribosomes in our model, we consider all possible states of elongating ribosomes (C0, C1 and C2 in Figure 5A) but we do not vary the total number of ribosomes. The reviewer is correct that the free (non-elongating) ribosomes can certainly affect translation efficiency, predominantly the initiation rate of transcripts. We have now extended our discussion to cover this topic about the coupling between translation initiation and elongation.*

In sum, while the author found very interesting correlations but those correlations and observation does not fully support very strong conclusion such as "Our findings provide direct evidence that codon bias is exploited to yield dynamic changes in translation efficiency, and that rare codons, rather than persisting passively in genomes due to reduced selection for translation efficiency, may be actively used to modulate translation rates." For this strong conclusion, I honestly think that tRNA level need to be perturbed (technically very hard) and evidence very clear arrest or lost proliferation ability.

*We thank the reviewer for this comment, and we have certainly thought about such perturbation experiments before. However, as pointed out by the reviewer, these are very challenging from a technical point of view and furthermore, it would be very difficult to find an experiment where we could properly modulate the global levels of tRNAs without affect other cellular functions. Our data though provides multiple lines of evidence that there is a correlation between the elongation efficiency of rare codons and the proliferative state of cells. We have evaluated possible mechanisms, such as the regulation of individual tRNAs and also the presence of tRNA modifications presumably capable of affecting the translation of rare codons (new analysis in Figure 4D, E). We did not find any evidence that these mechanisms, which have already been put forward in the literature, play a role in our observations. Therefore, another mechanism appears to be at work. In our manuscript we propose an alternative hypothesis for how codon-specific effects can emerge when tRNA levels change globally. While we agree that further studies need to be performed to firmly validate this mechanism, we believe our solid data demonstrating that mRNAs enriched in rare codons are dynamically regulated in proliferating cells as well as the novel mechanism that we propose will be of great interest to the field of translational control.*

**Second round of review**

**Reviewer 1**

The authors investigate a very intriguing topics and postulate the rare codon can potentially provide a regulatory way to increase translation during cell proliferation.

The idea and concepts are extremely interesting however some of the experimental design can be improved and more important the conclusions need to have clear experimental support.

My mayor concern is the model that they propose. I think, that the model is what "the field" is expecting to find, based on the growing evidence that tRNA can regulate gene expression. However, I do not see any experimental data supporting the idea that tRNA levels increase during proliferation and that increased affects translation (Fig. 5D). Actually, their sequencing data (tRNA-seq) does not show any major change in tRNA level (Fig. 4C). While the authors correctly mentioned, as they have not used spikeIN molecules, global changes in tRNA cannot be addressed with this experiment. Therefore, this data does not provide any evidence in global changing of tRNA. However, the northern-blot in Figure 4F should not have this limitation and therefore, if global level of tRNAs are changing in proliferating cells, we can expect to see it in the northern-blot. However, several controls (at least loading controls, U6, or other small RNAs) are required to be able to conclude that tRNA level increase the proliferating cells.

The fact that is not clear whether tRNA level change or not, then the conclusion that can raise from the computational model are limited.

There are few references that can help the author writing the discussion or introduction thinking in the potential regulatory role of tRNA thinking about the tRNA charged ratio.

In this review the authors discussed the idea of role of the tRNA-ready-to-go:

Rak R, Dahan O, Pilpel Y. 2018. Annual Review of Cell and Developmental Biology.

Repertoires of tRNAs: the couplers of genomics and proteomics.

In this one the authors show that tRNA charged ratio correlates with the regulatory effect of the codon on mRNA stability.

Wu Q, Medina S, Kushawah G, DeVore M, Castellano L, Hand J, Wright M, Bazzini A. 2019. eLife.

Translation affects mRNA stability in a codon-dependent manner in human cells.

In this one, the author measure the tRNA charge ratio.

Frumkin I, Schirman D, Rotman A, Li F, Zahavi L, Mordret E, Asraf O, Wu S, Levy SF, Pilpel Y. 2017. Molecular Cell.

Gene architectures that minimize cost of gene expression.

And in this one, the authors also investigate the regulatory role of tRNA

Frumkin I, Lajoie MJ, Gregg CJ, Hornung G, Church GM, Pilpel Y. 2018. PNAS.

Codon usage of highly expressed genes affects proteome-wide translation efficiency.

Globally, I find the work i interesting and novel, however their results do not support their strong conclusions.

**Reviewer 2**

**Authors Response**

**Point-by-point responses to the reviewers’ comments:**

Reviewer #1:

The authors investigate a very intriguing topic and postulate the rare codon can potentially

provide a regulatory way to increase translation during cell proliferation.

The idea and concepts are extremely interesting however some of the experimental design can be

improved and more important the conclusions need to have clear experimental support.

My major concern is the model that they propose. I think that the model is what "the field" is

expecting to find, based on the growing evidence that tRNA can regulate gene expression.

However, I do not see any experimental data supporting the idea that tRNA levels increase

during proliferation and that increased affects translation (Fig. 5D).

*We do not share the reviewer’s opinion that ‘the model is what “the field” is expecting to find’.*

*Large efforts are currently dedicated to map translational responses in many systems, and the*

*main aim of our work was to assess experimentally, using a variety of orthogonal methods,*

*whether codon bias affects translation in cells growing at different proliferation rates. Of course,*

*having established this impact immediately prompts the question of what the underlying*

*mechanism may be. Here we have assessed a few models that have been proposed to explain*

*how codon usage can impact protein output, specifically through variation in the abundance of*

*individual tRNAs or in tRNA modifications. We did not find support for these models in our*

*data. An alternative model, also proposed before, is that variations in a global resource, such as*

*total ready-to-translate tRNA levels, could differentially impact distinct transcripts. We think*

*that it is rather such a model, that involves removal of a translation bottleneck experienced by*

*transcripts with rare codons in cells with low proliferation rate, that can best explain our data.*

*We explored the behavior of this model, showing that the sensitivity of decoding rate to the*

*availability of ready-to-translate tRNAs is consistent with the boost in translation of transcripts*

*enriched in rare codons that we observe experimentally. This scenario was suggested by a*

*previous study that found tRNA levels to be 2-fold higher in the G2 phase of the cell cycle*

*compared to the other phases [1]. We did not find such a difference comparing cells grown in*

*10% vs 1% FCS. This may be because our system does not exhibit such a difference or because*

*the small difference that we can expect (~15% change assuming the generality of the yeast*

*measurement and the 10-15% difference in G2 vs G1 cells in 10% vs 1% FCS conditions) is not*

*detectable with current methods. However, a similarly higher sensitivity in decoding rate of rare*

*codons compared to common codons can be inferred with respect to the rate of tRNA charging.*

*This latter scenario is suggested by our GSEA analysis of the gene expression data and the*

*measurement that we obtained for a specific tRNA. Thus, while resolving the precise kinetic*

*parameter that underlies increased availability of tRNAs awaits further work and improved*

*measurement methods, what our study indicates is that the translation effects that we observe are*

*more consistent with variations in a global factor (such as global ready-to-translate tRNA*

*availability) than a codon/tRNA-specific factor.*

Actually, their sequencing data (tRNA-seq) does not show any major change in tRNA level (Fig.

4C). While the authors correctly mentioned, as they have not used spikeIN molecules, global

changes in tRNA cannot be addressed with this experiment. Therefore, this data does not provide

any evidence in global changing of tRNA. However, the northern-blot in Figure 4F should not

have this limitation and therefore, if global level of tRNAs are changing in proliferating cells, we

can expect to see it in the northern-blot. However, several controls (at least loading controls, U6,

or other small RNAs) are required to be able to conclude that tRNA levels increase in

proliferating cells. The fact that it is not clear whether tRNA levels change or not, then the

conclusion that can raise from the computational model are limited.

*As we mentioned above, we in fact started from observations that translation components have a*

*specific cell cycle dynamics, accumulating in G2/M compared to G1. Although absolute*

*quantification of RNA abundance is not trivial, there are some studies where this has been done,*

*for rRNA [2] and tRNA [1]. The latter study specifically suggested that tRNA levels are ~2-fold*

*higher in G2 compared to G1. Thus, we did not think that this issue would be disputed, and we*

*put our efforts into additional mechanisms such as tRNA charging (Fig. 4).*

*We ideally would like to employ a “genome-wide” approach for tRNA quantification, rather than*

*gene-by-gene using “loading controls”, because the typical loading controls for northern blots*

*may also change in abundance dependent on the cell cycle phase or proliferation rate of cells.*

*However, we do not think that a satisfactory approach to genome-wide quantification of absolute*

*tRNA quantification is available. Assuming that the quantification from Ref. [1] is accurate, the*

*10-15% difference in the proportion of G2-phase cells between our culture conditions (Fig. 3D)*

*would result in ~15% difference in the amount of tRNAs extracted from similar numbers of*

*cells. Prompted by this comment of the reviewer we did make an attempt, by isolating total RNA*

*from similar numbers of cells grown in 1% vs. 10% FCS, quantifying RNA amount by riboGreen and also quantifying relative abundance of RNAs in the tRNA size range with a*

*bioanalyzer. However, the variation in total RNA among replicates of the same condition was*

*much larger than 15% and therefore, we could not draw reliable conclusions from these*

*measurements.*

*On the other hand, similar to what has been reported in the previously-mentioned study [1], we*

*also found that various aspects of tRNA metabolism are upregulated in proliferating cells (Fig.*

*4A), and at least for one specific tRNA, northern blot measurements indicate somewhat*

*increased charging rate (Fig. 4F, G). Thus. of the tRNA-dependent models that have been*

*proposed so far, we think that our data is most consistent with a model involving a global change*

*in the translation capacity. This could rely on increased total tRNA levels, or on other general*

*factors such as increased tRNA charging. We revised the text to make these aspects more clear.*

There are few references that can help the author writing the discussion or introduction thinking

in the potential regulatory role of tRNA thinking about the tRNA charged ratio.

In this review the authors discussed the idea of role of the tRNA-ready-to-go:

Rak R, Dahan O, Pilpel Y. 2018. Annual Review of Cell and Developmental Biology.

Repertoires of tRNAs: the couplers of genomics and proteomics.

In this one the authors show that tRNA charged ratio correlates with the regulatory effect of the

codon on mRNA stability.

Wu Q, Medina S, Kushawah G, DeVore M, Castellano L, Hand J, Wright M, Bazzini A. 2019.

eLife.

Translation affects mRNA stability in a codon-dependent manner in human cells.

In this one, the authors measure the tRNA charge ratio.

Frumkin I, Schirman D, Rotman A, Li F, Zahavi L, Mordret E, Asraf O, Wu S, Levy SF, Pilpel

Y. 2017.

Molecular Cell. Gene architectures that minimize cost of gene expression.

And in this one, the authors also investigate the regulatory role of tRNA

Frumkin I, Lajoie MJ, Gregg CJ, Hornung G, Church GM, Pilpel Y. 2018. PNAS.

Codon usage of highly expressed genes affects proteome-wide translation efficiency.

*We thank the reviewer for pointing out these interesting references. We have incorporated the*

*most pertinent work in our introduction and discussion.*

Globally, I find the work i interesting and novel, however their results do not support their strong

conclusions.

*The reviewer is not specific regarding which conclusions are not strongly supported. Based on*

*the previous comments, we infer that the reviewer refers to the specific model that we analyzed.*

*We made it more clear now that this is one form that a model of varying translation capacity*

*could take, but that we think that our data is most consistent with a global as opposed to a*

*codon/tRNA-specific factor. What we wanted to emphasize with this model is how a global*

*change in a translation component can differentially affect transcripts with different codon bias,*

*and especially how it can lead to a larger translation boost of transcripts enriched in rare codons.*

*Most literature, including some of the papers mentioned above by the reviewer, focuses on*

*optimal codons being needed for high expression, whether by avoiding translation bottlenecks or*

*by increasing mRNA stability. Our data indicate that rare codons may not simply be deleterious*

*for translation, but rather support a more nuanced dynamics of translation, which we*

*independently verified using both genome-wide approaches and synthetic constructs. We argue*

*that this finding is important for drawing the attention of the field to the manner in which rare*

*codons are involved in translational control.*

*Cited work*

*1. Frenkel-Morgenstern M, Danon T, Christian T, Igarashi T, Cohen L, Hou Y-M, et al. Genes*

*adopt non-optimal codon usage to generate cell cycle-dependent oscillations in protein levels.*

*Mol Syst Biol. 2012;8:572.*

*2. Grummt I. Life on a planet of its own: regulation of RNA polymerase I transcription in the*

*nucleolus. Genes Dev. 2003;17:1691–702.*
